# Targeting KDM4B that coactivates c-Myc-regulated metabolism to suppress tumor growth in castration-resistant prostate cancer

**DOI:** 10.7150/thno.58729

**Published:** 2021-06-26

**Authors:** Meng-Jen Wu, Chih-Jung Chen, Ting-Yu Lin, Ying-Yuan Liu, Lin-Lu Tseng, Mei-Ling Cheng, Chih-Pin Chuu, Huai-Kuang Tsai, Wen-Ling Kuo, Hsing-Jien Kung, Wen-Ching Wang

**Affiliations:** 1 Institute of Molecular and Cellular Biology and Department of Life Science, National Tsing-Hua University, Hsinchu 30013, Taiwan.; 2 Department of Pathology and Laboratory Medicine, Taichung Veterans General Hospital, Taichung 40705, Taiwan.; 3 School of Medicine, Chung Shan Medical University, Taichung, 40201, Taiwan.; 4 Department of Biomedical Sciences, College of Medicine, Chang Gung University, Taoyuan 333, Taiwan.; 5 Institute of Cellular and System Medicine, National Health Research Institutes, Miaoli 35053, Taiwan.; 6 Institute of Information Science, Academia Sinica, Taipei, 11529, Taiwan.; 7 Division of Breast Surgery, General Surgery, Department of Surgery, Chang Gung Memorial Hospital Linko Medical Center, Taoyuan 333, Taiwan.; 8 Graduate Institute of Cancer Biology and Drug Discovery, Taipei Medical University, Taipei 110, Taiwan.; 9 Department of Biochemistry and Molecular Medicine, University of California Davis School of Medicine, University of California Davis Cancer Centre, Sacramento, CA 95817, USA.

**Keywords:** Castration-resistant prostate cancer, KDM4B, c-Myc, histone demethylase, metabolic rewiring

## Abstract

**Rationale:** The progression of prostate cancer (PCa) to castration-resistant PCa (CRPC) despite continuous androgen deprivation therapy is a major clinical challenge. Over 90% of patients with CRPC exhibit sustained androgen receptor (AR) signaling. KDM4B that removes the repressive mark H3K9me3/2 is a transcriptional activator of AR and has been implicated in the development of CRPC. However, the mechanisms of KDM4B involvement in CRPC remain largely unknown. Here, we sought to demonstrate the molecular pathway mediated by KDM4B in CRPC and to provide proof-of-concept evidence that KDM4B is a potential CRPC target.

**Methods:** CRPC cells (C4-2B or CWR22Rv1) depleted with KDM4B followed by cell proliferation (*in vitro* and xenograft), microarray, qRT-PCR, Seahorse Flux, and metabolomic analyses were employed to identify the expression and metabolic profiles mediated by KDM4B. Immunoprecipitation was used to determine the KDM4B-c-Myc interaction region. Reporter activity assay and ChIP analysis were used to characterize the KDM4B-c-Myc complex-mediated mechanistic actions. The clinical relevance between KDM4B and c-Myc was determined using UCSC Xena analysis and immunohistochemistry.

**Results:** We showed that KDM4B knockdown impaired CRPC proliferation, switched Warburg to OXPHOS metabolism, and suppressed gene expressions including those targeted by c-Myc. We further demonstrated that KDM4B physically interacted with c-Myc and they were co-recruited to the c-Myc-binding sequence on the promoters of metabolic genes (*LDHA*, *ENO1*, and *PFK*). Importantly, KDM4B and c-Myc synergistically promoted the transactivation of the *LDHA* promoter in a demethylase-dependent manner. We also provided evidence that KDM4B and c-Myc are co-expressed in PCa tissue and that high expression of both is associated with worse clinical outcome.

**Conclusions:** KDM4B partners with c-Myc and serves as a coactivator of c-Myc to directly enhance c-Myc-mediated metabolism, hence promoting CRPC progression. Targeting KDM4B is thus an alternative therapeutic strategy for advanced prostate cancers driven by c-Myc and AR.

## Introduction

Prostate cancer (PCa) is now the sixth leading cause of cancer-related deaths in men worldwide, ranking first and second in the UK and the United States, respectively 1. Androgen deprivation therapy by surgery or hormonal castration has been the standard treatment for androgen receptor (AR)-driven PCa since the 1970s 2. Despite an effective response that could last a few years, the majority of patients develop a more aggressive form of cancer, referred to as castration-resistant PCa (CRPC) carrying the sustained or even increased activation of AR activity. Several mechanisms underlying the development of CRPC have been described, including the generation of constitutively active forms of AR by various mechanisms (including AR amplification, AR mutations, and the expression of AR splice variants) 3-6 and/or the upregulation of CYP17A1 that increases intratumoral androgen synthesis 7. The FDA-approved, next-generation AR antagonist enzalutamide and the CYP17A1 inhibitor, abiraterone, have significantly improved the outcomes for patients with CRPC 7, 8. Although they effectively alleviate symptoms and prolong survival, most patients eventually develop resistance, leading to death within a short period of time; a relapse usually takes place after 1-2 years of treatment and within 4-6 months for advanced cases (post-chemotherapy) 7, 8, emphasizing the urgent need for more effective treatment.

Understanding the molecular mechanisms underlying the development of CRPC provides a basis for identifying candidate therapeutic targets beyond the next-generation AR signaling inhibitors. AR spliced variants that display gain of function or even function as constitutively active forms (for instance, AR-V7) contribute to disease progression and therapeutic resistance 9. Detecting and targeting AR variants provides a strategy to overcome AR-V activity in the clinic 10, 11.

c-Myc, a potent oncogenic transcription factor, is deregulated in many human malignancies. In particular, c-Myc overexpression has been linked to aggressive, poorly differentiated tumors, including CRPC 12, 13. The inhibition of c-Myc, which regulates the expression and activity of AR and active AR variants, sensitizes enzalutamide-resistant CRPC cells and tumors in patient-derived xenograft models 14. By the use of c-Myc transgenic murine models, high expression of c-Myc drives prostatic intraepithelial neoplasia into invasive adenocarcinoma, similar to that seen in human PCa 15. Conditional transgenic mouse models also shows that c-Myc is a key factor in the initiation and maintenance of tumorigenesis 16. Interestingly, the introduction of N-Myc (another member of the Myc family) and AKT1 into epithelial precursor cells is sufficient to recapitulate human prostate adenocarcinoma and the hormone-insensitive neuroendocrine phenotype in an engineered mouse model 17. Based on the well-established role of c-Myc in cancer initiation and progression, various strategies have been developed to suppress its activity for clinical intervention. As c-Myc is generally considered “non-druggable”, several strategies have been developed including targeting c-Myc for degradation (aurora kinase inhibitor work) or targeting c-Myc-binding partners crucial for its transcriptional activity as well as its c-Myc-mediated effectors 13, 18. For instance, the inhibition of the BET domain-containing Brd4, a c-Myc regulator, by a small compound, JQ1, has been found to curb tumor growth 19-21. However, this is only expected to be effective for patients harboring Brd4 in c-Myc-driven tumors.

The KDM4 histone lysine demethylase family has been identified as another potential target for tumor therapy 22, 23. Members of the KDM4/JMJD2 family (KDM4A-KDM4D), which catalyze the demethylation activity of methylated lysine sites on H3, contain a conserved Jumonji C catalytic domain that requires α-ketoglutarate (α-KG), Fe(II), and molecular oxygen as cofactors 23, 24. They exhibit substrate activity toward two methyl marks (H3K9me3/me2 and H3K36me3/me2) 25, 26. H3K9me3 is enriched in heterochromatic areas and silences genes when covering cis-regulatory elements, and H3K36me3 is considered a transcriptional elongation-related mark 27, 28. Three full-length members (KDM4A/4B/4C) share a conserved active-site framework in the JmjN-JmjC domain but only >50% sequence identity in the DNA-binding domains (two plant homeodomains and two Tudor domains). Despite the homology among KDM4A, KDM4B, and KDM4C, they have non-redundant functions and trigger unique signaling pathways involved in diverse cellular processes 23. KDM4A and KDM4B interact with AR and are often overexpressed in severe, advanced forms of PCa 29-31. Furthermore, KDM4A coactivates E2F1 to promote the glycolytic switch favored by PCa cells (LNCaP) 32. KDM4C contributes to pluripotency and stemness 33. KDM4C expression is reduced during acinar morphogenesis of human RWPE-1 prostate epithelial cells 34. KDM4B is induced by hypoxia, a condition associated with castration resistance 35, and promotes cell proliferation in LNCaP 31. Moreover, KDM4B promotes prostate tumorigenesis via cell cycle 36 and regulates the alternative splicing of AR for generating AR-V7 in response to androgen deprivation 37. Overexpression of KDM4B transactivates AR-dependent c-Myc expression and contributes to resistance to enzalutamide through recruitment of AR to the site of *MYC* gene enhancer 38. However, the role of KDM4B in CRPC progression and metabolic regulation remains largely unknown.

In this study, we show that KDM4B knockdown effectively impairs the viability of AR-positive CRPC cells as well as the tumor growth in xenografts. The depletion of KDM4B resulted in a switch from anaerobic glycolysis to oxidative phosphorylation (OXPHOS) in AR-positive CRPC cells. Importantly, we found that KDM4B directly interacts with c-Myc and co-regulated c-Myc target genes, such as *LDHA*. We also show that KDM4B is highly expressed in PCa and is related to poor survival. As a dual coactivator of c-Myc and AR, KDM4B represents a promising new therapeutic target against CRPC.

## Materials and Methods

### Cell culture

PZ-HPV-7, RWPE-1, LNCaP, CWR22Rv1, PC-3, DU-145 and HEK293T cell lines were from American Type Culture Collection (ATCC, Manassas, VA, USA). PZ-HPV-7 and RWPE-1 were cultured in Keratinocyte Serum Free Medium (K-SFM) (Thermo, Waltham, MA, USA) supplemented with bovine pituitary extract (Thermo) and human recombinant epidermal growth factor (Thermo). LNCaP, CWR22Rv1, PC-3 and DU-145 were cultured in RPMI 1640 medium (Thermo) containing 10% fetal bovine serum (FBS) (Hyclone, Logan, UT, USA). HEK293T were cultured in Dulbecco's Modified Eagle Medium (DMEM) (Thermo) containing 10% FBS. LNCaP-derived C4-2 and C4-2B cell lines 39 were cultured in RPMI 1640 medium containing 10% FBS. Cells were incubated in a humidified incubator with 5% CO_2_ at 37 °C.

### Antibodies, plasmids and reagents

Rabbit anti-KDM4B (Cat. No. A301-478A) was purchased from Bethyl Laboratories (Montgomery, TX, USA). Rabbit anti-HA-tag (Cat. No. 3724S) was purchased from Cell Signaling Technology (Danvers, MA, USA). Rabbit Anti-c-Myc (Cat. No. ab152146) was purchased from Abcam (Cambridge, UK). Mouse anti-Flag (Cat. No. F1804) and anti-β-Actin (Cat. No. A5441) were purchased from Sigma-Aldrich (St. Louis, MO, USA). Rabbit anti-IgG (Cat. No. PP64) was purchased from Millipore (Billerica, MA, USA). Mouse anti-IgG (Cat. No. sc-2025) was purchased from Santa Cruz Biotechnology (Dallas, TX, USA). Rabbit anti-H3K9me3 (Cat. No. 39765) was purchased from Active Motif (Carlsbad, CA, USA). Rabbit anti-c-Myc (Cat. No. GTX103436) were purchased from GeneTex (Irvine, CA, USA). Goat anti-rabbit (Cat. No. 926-32211) was purchased from LI-COR (Lincoln, NE, USA). Goat anti-mouse (Cat. No. A21036) was purchased from Thermo. Lentiviral vector pLKO.1-control (LKO), pLKO-shKDM4A (sh4A#1, TRCN0000234912 and sh4A#2, TRCN0000234914), pLKO.1-shKDM4B (sh4B#1, TRCN0000018016 and sh4B#2, TRCN0000379460) and pLKO.1-shKDM4C (sh4C#1, TRCN0000235047 and sh4C#2, TRCN0000235048) plasmids were purchased from The RNAi Consortium (TRC) library in Taiwan. The pCMV-HA-JMJD2B (Addgene #24181) expression plasmid and the pBV-Luc MBS1-4 (Addgene #16564) luciferase plasmid were purchased from Addgene (Watertown, MA, USA). The truncated pCMV-HA-JMJD2B (ΔN470, ΔC180, ΔC290, and ΔC460), pCMV-HA-JMJD2B-H189A, and specific KDM4B shRNA-resistant plasmids were described previously 40. The full-length, N- and C-terminal truncated constructs of c-Myc were constructed through the insertion of PCR fragments from the pBS human c-Myc plasmid (Addgene #14971) with PCR by specific primers (listed in Table S1) into pCMV-Flag, yielding Flag-c-Myc variants. The pNL1.1.TK[Nluc/TK] vector was purchased from Promega (Madison, WI, USA). The pGL3-LDHA-Luc reporter construct was generated through the insertion of a 1.4-kb PCR fragment corresponding to -1,034 bp‒+380 bp of *LDHA* into the pGL3-luc vector (Promega). MYC siRNA (Cat. No. L-003282-02-0005) and PDHA1 siRNA (Cat. No. L-010329-00-0005) were purchased from Dharmacon (Lafayette, CO, USA).

### RNA interference

Recombinant lentivirus was generated by transfection with lentiviral vector encoding pLKO.1 or a shRNA that specifically targets the* KDM4B* gene and packaging vectors pLP1, pLP2, and pLP/VSVG (Thermo) in 293T cells. Virus particles in the supernatant were harvested after 76 h. PCa cells were transduced with pLKO.1 or a shRNA viral particle, followed by selection with puromycin for 48 h. The knockdown efficiency was confirmed using immunoblotting analysis.

### Immunoblotting

Total cell lysates were obtained by lysing cells with RIPA buffer supplemented with protease inhibitor (Santa Cruz) and PhosSTOP (Sigma). Appropriate amounts of lysates were fractionated by SDS-PAGE on 10% acrylamide gel and then transferred onto PVDF membrane with 0.22 µm pore size (PALL, Port Washington, NY, USA). Membrane was incubated with diluted primary antibody (KDM4B, 1:2000; c-Myc, 1:1000; β-Actin, 1:50000; HA, 1:1000; Flag, 1:5000) at 4 °C overnight and then probed with secondary antibodies (Rabbit, 1:20000; Mouse, 1:20000) conjugated with fluorescence. Odyssey Infrared Imaging System (LI-COR Biosciences, Lincoln, NE, USA) was used to detect the level of fluorescence.

### Quantitative RT-PCR (qRT-PCR)

Total RNA was extracted with TRIzol reagent (Thermo) and the concentration of total RNA was measured with Nano-DropTM Spectrophotometers (Thermo). Appropriate amounts of total RNA were reverse transcribed using the SuperScript III Reverse Transcriptase (Thermo), dNTP (Genedirex, Las Vegas City, NV, USA), and random primers (Thermo). Real-Time PCR was performed with the SensiMixTM SYBR® Hi-ROX Kit (Bioline, Taunton, MA, USA) and primers (listed in Table S1) using ABI StepOnePlus Real-Time PCR System (Thermo).

### Cell proliferation assay

For cell counting, cells (LNCaP, 1×10^5^ cells/well; C4-2B, 1×10^5^ cells/well; CWR22Rv1, 2×10^5^ cells/well) were seeded in 6-well plate and incubation at 37 °C overnight. Cell number was measured every day for a total of 4 days using Countess II FL Automated Cell Counter (Thermo). For MTT assay, cells (LNCaP, 3×10^3^ cells/well; C4-2B, 3×10^3^ cells/well; CWR22Rv1, 6x10^3^ cells/well) were seeded in 96-well plates and incubate at 37 °C overnight. Cell viability was measured every 1 day for a total of 4 days by MTT (Thermo) using CLARIOstar® Microplate Reader (BMG Labtech, Ortenberg, Germany).

### Immunoprecipitation (IP)

Cell pellets were collected and lysed in IP lysis buffer (50 mM Tris-HCl (pH 7.4), 150 mM NaCl, 0.5% NP40, and protease inhibitor) at 4 °C for 1.5 h. The equal cell lysate, primary antibody (1 μg), and 10 μl PureProteome protein A/G magnetic beads (Millipore) were incubated together at 4 °C overnight with gentle rocking. The beads were washed three times with IP wash buffer (137 mM NaCl, 2.7 mM KCl, 10 mM Na_2_HPO_4_, 1.8 mM KH_2_PO_4_, 0.1% Tween 20, pH 7.4). The bound protein complexes were then eluted by IP lysis buffer at 85 °C for 10 min. The complexes were fractionated on SDS-PAGE for immunoblotting analysis.

### Microarray

Global gene expression analysis of C4-2B cells (LKO *vs.* sh4B#1) (GEO number: GSE147481) was performed at National Health Research Institutes (Miaoli, Taiwan), using Affymetrix GeneChip Human Gene 2.0 ST Array (Affymetrix, Santa Clara, CA, USA). Gene annotation and pathway of KEGG were done by DAVID Database (https://david.ncifcrf.gov).

### Measurement of ECAR and OCR

The cellular metabolic function was carried out using Seahorse XFp Analyzer (Agilent, Santa Clara, CA, USA) according to the manufacturer's instruction. Briefly, cells (C4-2B, 2×10^4^ cells/well; CWR22Rv1, 4×10^4^ cells/well) were seeded in 8-well Seahorse plates which were coated with Poly-D-Lysine and incubated at 37 °C overnight. For mitochondrial respiration analysis, cells were incubated in XF assay media supplemented with 2% FBS, 1 mM sodium pyruvate, 2 mM L-glutamine, and 10 mM glucose and sequentially injected with 1 μM oligomycin, FCCP (0.5 μM for C4-2B; 1 μM for CWR22Rv1), and 50 μM rotenone/antimycin A. For glycolysis analysis, cells were incubated in XF assay media supplemented with 2% FBS and 2 mM L-glutamine and then injected with 10 mM glucose, 1 μM oligomycin, and 50 mM 2-DG in sequence. All chemicals were purchased from Sigma.

### PDH activity

Cells were seeded on 6-cm dishes (1×10^6^ cells) and cultured at 37 °C. After 48 h, cell pellets were collected and the cellular PDH activity was measured using Pyruvate Dehydrogenase Activity Colorimetric Assay Kit (Biovision, Milpitas, CA, USA) according to the manufacturer's instructions.

### ROS detection

The cellular ROS level was detected using the 2',7'-dichlorodihydrofluorescein diacetate (H2DCFDA, Thermo) assay as previously described 41. In brief, cells (5×10^5^ cells/well) were seeded onto glass slides in 6-well plates and incubates at 37 °C overnight. Cells were washed with PBS, followed by incubation with H2DCFDA (50 μM) at 37 °C for 30 min. The cells were washed, paraformaldehyde fixed, permeabilized and stained with DAPI to label nuclei. The fluorescence signals of H2DCFDA were detected by ZEISS LSM800 confocal microscopy (ZEISS, Oberkochen, Germany) and the images were analyzed with Zen2.3 software and ImageJ.

### Analysis of metabolites by liquid chromatography mass spectrometry (LC-MS)

Metabolite extraction and LC/MS analysis was performed as previously described 42. Samples were subjected to chromatographic separation on an Acquity HSS T3 reversed-phase C18 column (2.1×100 mm, particle size of 1.8 mm; Waters Corp., Milford, MA, USA), using a 1200 rapid-resolution system (Agilent Technologies, Santa Clara, CA, USA), followed by subsequent mass spectrometric analysis.

### Luciferase assay

Cells were seeded on 24-well plates (C4-2B, 7×10^4^ cells/well; CWR22Rv1, 1×10^5^ cells/well) and incubated at 37 °C overnight. The plasmids of pBV-Luc MBS1-4, pGL3-LDHA-Luc, pCMV-HA-mock, pCMV-HA-KDM4B, pCMV-flag-c-Myc, pCMV-HA-KDM4B-H189A, and internal control reporter β-galactosidase were transfected into cells using Lipofectamine 2000 (Thermo). After 24 h, the promoter activities were measured using Beta-Glo/One-Glo Assay System (Promega).

### Chromatin immunoprecipitation (ChIP) assay

ChIP assay was performed as described previously 40. In brief, cells were crosslinked with 1% formaldehyde and the crosslinked chromatin was fragmented to 200-500 bp by sonication. The appropriate amounts of lysates were incubated with Magna ChIP Protein G Magnetic beads (Millipore) and specific antibodies (5 μg) at 4 °C overnight with gentle rocking. The ChIP complexes were eluted and analyzed by qRT-PCR (primer sequences listed in Table S1). Fold enrichment was calculated by the ΔΔCt method.

### ChIP-seq analysis of KDM4B-bound and c-Myc-bound targets in K562

The ChIP-seq data of KDM4B (ID: ENCSR642VZY) and c-Myc (ID: ENCSR000EGJ) in K562 were obtained from ENCODE (https://www.encodeproject.org). Peaks were annotated using Homo sapiens (human) genome assembly GRCh38 (hg38).

### Xenograft

C4-2B and CWR22Rv1 cells (1×10^6^ cells) were suspended in culture medium and mixed with equal Matrigel Matrix (BD, Franklin Lakes, NJ, USA). The mixtures were implanted subcutaneously into six- to eight-week-old male Balb/c nu/nu mice. One week after implantation, the tumor volume was monitored at the indicated time points. The tumor volume was calculated using formula: length (L)×width (W)×height (H)×0.52. Statistical analysis was conducted as described below. All mice were sacrificed after the end point. The animal studies were approved by National Tsing Hua University Institutional Animal Care and Use Committee (approval number: NTHU- IACUC-10478) and carried out under the institutional guidelines with animal welfare standards.

### UCSC Xena analysis

Expression profiles and Kaplan-Meier survival curves of KDM4B and c-Myc were performed from UCSC Xena platform that consists of public genomic data (https://xenabrowser.net). Gene expression profiles were compared between TCGA prostate cancer samples (n = 495) and GTEx healthy prostate tissue samples (n = 100). The Kaplan-Meier overall survival analysis was conducted within a 150-month period using data extracted from TCGA Prostate Cancer (PRAD). The expression profiles and Kaplan-Meier plots were generated and analyzed using Graphpad Prism 7.

### Immunohistochemistry (IHC)

Two consecutive paraffin-embedded human prostate cancer tissue micro-arrays (102 cases) were purchased from Pantomics (#PRC1021; Richmond, CA, USA). The biopsies were deparaffinized, antigen retrieved with citrate buffer (pH 6.0), and stained with anti-KDM4B (GeneTex, #GTX53804, 1:200) or c-Myc (GeneTex, #GTX103436, 1:200) primary antibodies using Novolink Polymer Detection Systems (Leica, Wetzlar, Germany) according to the manufacturer's instruction. The signals of KDM4B and c-Myc in the tumor nucleus were scored as follows: 0, no positive signal; 1+, weak partial staining; 2+, weak to moderate intensity; 3+, strong intensity. The correlation between KDM4B and c-Myc were analyzed with Chi-square test.

### Statistical analysis

For IHC assay, Chi-square test was performed to compare groups with categorical variables in IHC analysis. For other experiments, statistical analyses were performed by student's *t*-test using the GraphPad Prism software. A *p* value < 0.05 was considered statistically significant.

## Results

### KDM4B is crucial for the proliferation of AR-positive CRPC cells

**Figure 1:**
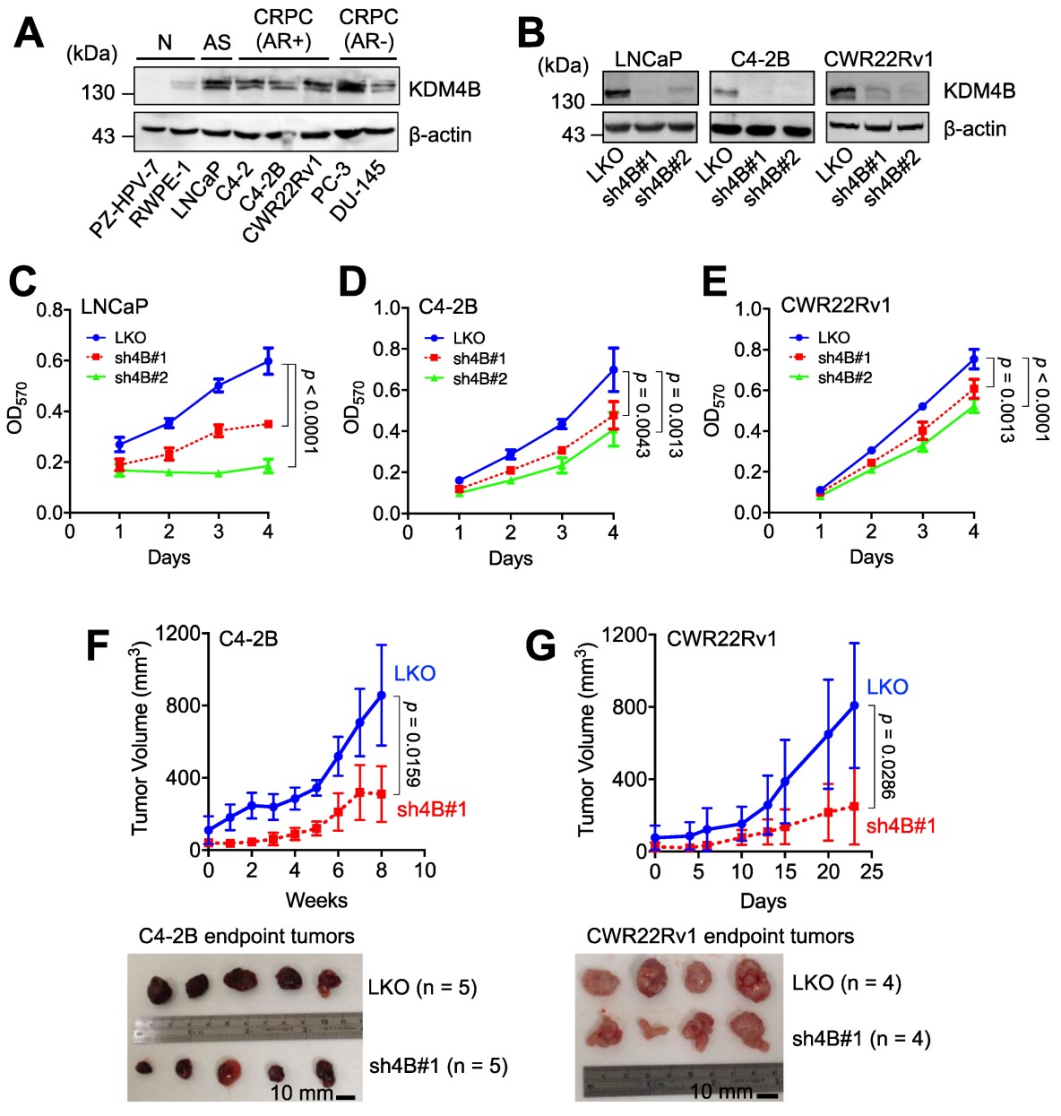
** KDM4B is crucial for the growth of AR-positive PCa cells. (A)** Expression of KDM4B in normal immortalized cells (RWPE-1 and PZ-HPV-7), and PCa cell lines (LNCaP, C4-2, C4-2B, CWR22Rv1, PC-3 and DU-145). AS, androgen sensitive; AR+, AR-positive; AR-, AR-negative; CRPC, castration-resistant PCa. **(B)** Generation of KDM4B-knockdown LNCaP, C4-2B, and CWR22Rv1 cells based on the lentivirus approach. Cells were infected with lentivirus carrying control pLKO.1 or a shKDM4B construct (sh4B#1 or sh4B#2). The knockdown efficiencies were confirmed by immunoblotting. β-Actin was the internal control. **(C-E)** MTT cell proliferation assay of control (LKO) and KDM4B-knockdown (sh4B#1 and sh4B#2) LNCaP **(C)**, C4-2B **(D)**, and CWR22Rv1 **(E)** cells at indicated time points. Data presented are means ± SD from three independent experiments. Statistical analysis of the differences between two groups was calculated by two-sided Student's *t*-test. **(F-G)** The depletion of KDM4B reduced the tumor growth in xenografts of C4-2B **(F)** and CWR22Rv1 **(G)** xenograft. LKO and KDM4B-knockdown (sh4B#1) cells were injected subcutaneously into the male BALB/c nude mice. The tumor volumes were measured at indicated time points. Scale bar, 10 mm. Data are presented as the mean ± S.D. Statistical significance was evaluated using Mann-Whitney U test.

We examined the expression of KDM4B in immortalized normal prostate epithelial cells (PZ-HPV-7 and RWPE-1), AR-positive PCa cells (LNCaP), AR-positive CRPC cells (C4-2, C4-2B, and CWR22Rv1), and AR-negative PCa cells (PC-3 and DU-145). LNCaP, three AR-positive CRPC lines, and two AR-negative CRPC lines exhibited higher levels of KDM4B than those in normal prostate epithelial cell lines (**Figure 1A**). To assess whether KDM4B is crucial for PCa cell proliferation, a lentiviral-based approach with the control (LKO) and two independent lines (sh4B#1 and sh4B#2) was utilized for KDM4B depletion in AR-positive LNCaP, C4-2B, and CWR22Rv1 cells (**Figure 1B**), resulting in significant reductions in cell proliferation [LNCaP: LKO *vs.* sh4B#1, *p* < 0.0001; LKO *vs.* sh4B#2, *p* < 0.0001; C4-2B: LKO *vs.* sh4B#1, *p* = 0.0043; LKO *vs.* sh4B#2, *p* = 0.0013; CWR22Rv1: LKO *vs.* sh4B#1, *p* = 0.0013; LKO *vs.* sh4B#2, *p* < 0.0001] (**Figure 1C-E and S1A-C**).

To substantiate the role of KDM4B in cell proliferation, we ectopically expressed KDM4B (4B) or a control (mock) in C4-2B. As compared with the control, KDM4B-expressing C4-2B exhibited a significantly increased level of cell proliferation (4B *vs.* mock, *p* = 0.0002) (**Figure S2E**). Comparable results were also obtained using the CWR22Rv1 model (*p* = 0.0001) (**Figure S2F**). Additionally, the introduction of a specific shRNA-resistant KDM4B (resi.) or a control (mock) vector into KDM4B-knockdown (sh4B#1 or sh4B#2) significantly restored the levels of cell proliferation (C4-2B: sh4B#1-resi. *vs.* sh4B#1-mock, *p* < 0.0001; sh4B#2-resi. *vs.* sh4B#2-mock, *p* = 0.0012; CWR22Rv1: sh4B#1-resi. *vs.* sh4B#1-mock, *p* = 0.0004; sh4B#2-resi. *vs.* sh4B#2-mock, *p* = 0.0001) (**Figure S2G-H**). Collectively, these data suggest that KDM4B was crucial to promote cell proliferation in CRPC cells.

We next determined whether KDM4B was required for CRPC growth using an animal xenograft model with CRPC cells (C4-2B and CWR22Rv1). Mice were subcutaneously injected with the control (LKO) or KDM4B-knockdown cells (sh4B#1). As shown in **Figure 1F-G**, KDM4B knockdown led to a significant impairment of tumor growth in C4-2B and CWR22Rv1 xenografts (*p* = 0.0159 and *p* = 0.0286, respectively). Together, our data suggested the notion that inhibition of KDM4B is a potential intervention strategy against CRPC.

### Knockdown of KDM4B reduces glycolytic flux and increases oxygen consumption

**Figure 2:**
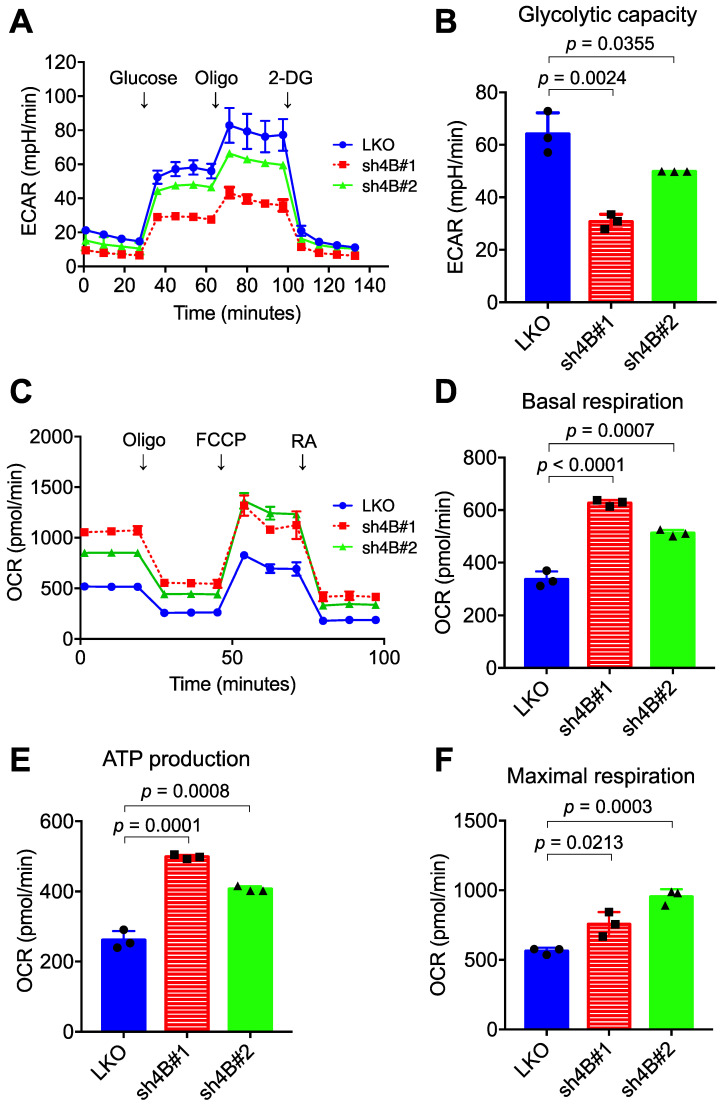
**Depletion of KDM4B alters the extracellular acidification rate (EACR) and oxygen consumption rate (OCR).** The ECAR **(A)** and OCR **(C)** of KDM4B-knockdown (sh4B#1 or sh4B#2) or control (LKO) C4-2B were measured by the Seahorse analyzer. For the ECAR assay, adherent cells were incubated at glucose-free and NaHCO_3_-free medium, followed by injection of 10 mM glucose, 1 µM oligomycin (Oligo), and 50 mM 2-DG. For the OCR assay, adherent cells were incubated at NaHCO_3_-free medium with subsequent injection of 1 μM oligomycin (Oligo), 0.5 μM FCCP, and 50 μM rotenone/antimycin A (RA). **(B)** The glycolytic capacity was plotted based on the ECAR assay** (A)**. **(D-F)** are metabolic parameters inferred from the OCR assay **(C)**, basal respiration** (D)**, ATP production **(E)**, and maximal respiration **(F)**. All data are presented as the mean ± S.D. and *p* values were calculated by two-sided Student's *t*-test.

To understand the molecular mechanism underlying KDM4B-dependent cell proliferation, the differential gene expression profile between LKO and sh4B#1 in C4-2B cells was analyzed by microarray assay (GEO number: GSE147481). Gene annotation and pathway mapping in KEGG revealed that a large number of down-regulated genes (n =109) in sh4B#1 cells are classified in metabolic pathways (**Figure S3A**). Of note, those genes contribute to glycolysis and pyruvate metabolism based on KEGG analysis (**Figure S3B**), implicating that KDM4B is crucial in energy metabolism, a hallmark in aggressive PCa for malignant transformation and growth 43. We therefore asked whether KDM4B was crucial for aberrant glycolytic metabolism in CRPC. LKO and KDM4B-knockdown cells were subjected to metabolic profiling using the Seahorse Analyzer. Interestingly, the extracellular acidification rate (ECAR) associated with glycolytic metabolism was significantly lower in KDM4B-knockdown C4-2B than in LKO (**Figure 2A-B**). By contrast, rates of oxygen consumption (OCR) were significantly higher in KDM4B knockdown cells at basal, ATP-linked, and maximal capacities than in LKO cells (**Figure 2C-F**). Likewise, there were comparable glycolytic (**Figure S4A-B**) and mitochondrial respiratory (**Figure S4C-F**) profiles using the CWR22Rv1 model.

We next monitored the metabolic profile in C4-2B LKO ectopically expressed with KDM4B (4B) or a control (mock) using the Seahorse Analyzer. As shown in **Figure S5A** and **S5B**, a significantly higher level of glycolytic capacity was found in LKO-4B than in LKO-mock (*p* = 0.0108). Additionally, restoration of KDM4B in KDM4B-knockdown C4-2B using a specific shRNA-resistant KDM4B vector (resi.) significantly rescued the glycolytic capacity as compared to that of the control (sh4B#1-resi. *vs.* sh4B#1-mock, *p* = 0.0306; sh4B#2-resi. *vs.* sh4B#2-mock, *p* = 0.0107) (**Figure S5A-B**). On the other hand, LKO-4B exhibited a significantly lower degree of mitochondrial respiratory function than did LKO-mock (**Figure S5C-F**). Restoration of KDM4B in KDM4B-knockdown C4-2B also significantly reduced its mitochondrial respiratory activity as compared with KDM4B-knockdown C4-2B (**Figure S5C-F**). Using the CWR22Rv1 model, there were comparable KDM4B-mediated metabolic changes (**Figure S6A-F**). Together, these results suggested that KDM4B enhances the glycolytic capacity while suppresses mitochondrial respiratory function in CRPC.

**Figure 3:**
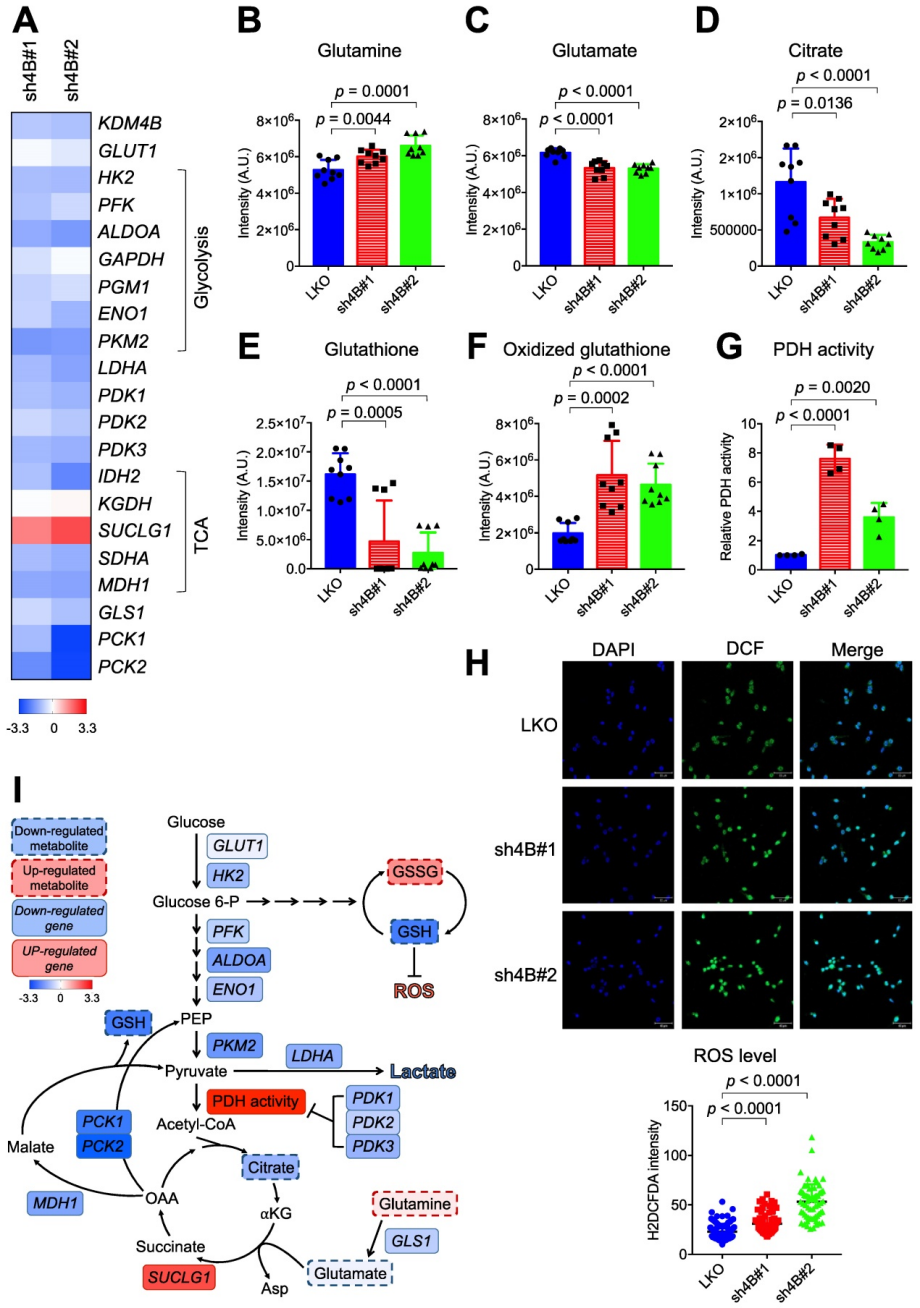
**KDM4B modulates energetic metabolism in C4-2B cells. (A)** qRT-PCR analysis of gene expression in KDM4B-knockdown (sh4B#1 and sh4B#2) C4-2B cells as a log2 fold change of that in control (LKO) cells. The upregulated genes were marked in red and the downregulated genes were in blue. The values are means of three independent experiments. **(B-F)** Analysis of cellular metabolites in the energy pathways for control and KDM4B-knockdown cells. Cells (LKO, sh4B#1, and sh4B#2) were cultured for 24 h. Glutamine **(B)**, glutamate **(C)**, citrate **(D)**, glutathione **(E)**, and oxidized glutathione **(F)** in lysates were analyzed by LC-MS analysis. Data are presented as the average of n = 9 replicates ± SD. **(G)** PDH activity of KDM4B-knockdown (sh4B#1 and sh4B#2) and control (LKO). Cells were cultured for 24 h and were harvested, followed by the measurement of PDH activity according to the manufacture's instruction (PDH Activity Colorimetric Assay Kit). **(H)** Analysis of cellular ROS using the H2DCFDA (DCF) assay. Cells were stained with DCF to label ROS (green) and DAPI to label nuclei (blue), followed by capturing images with ZEISS LSM800 confocal microscopy. Scale bar, 50 μm. The relative fluorescence intensity of cells was determined by Image J. Statistical analysis was assessed by Student's *t*-test.** (I)** A schematic diagram depicts the KDM4B-regulated metabolism to maintain redox and support tumor growth. Upregulation (red) or downregulation (blue) of genes from **(A)**, metabolites **(B-F)**, PDH activity **(G)**, and ROS **(H)** in KDM4B-knockdown cells as compared with the LKO cells is indicated. The colored key (blue-to-red) represent Log2 fold changes normalized by the average of KDM4B-knockdown cells. GLUT1, glucose transporter 1; HK2, hexokinase 2; PFK, phosphofructokinase; ALDOA, aldolase A; GAPDH, glyceraldehyde-3-phosphate dehydrogenase; PGM1, phosphoglucomutase 1; ENO1, enolase 1; PKM2, pyruvate kinase M2; LDHA, lactate dehydrogenase; PDK1/2/3, pyruvate dehydrogenase kinase 1/2/3; IDH2, isocitrate dehydrogenase 2; KGDH, α-ketoglutarate dehydrogenase; SUCLG1, succinyl-CoA synthetase; SDHA, succinate dehydrogenase A; MDH1, malate dehydrogenase 1; GLS1, glutaminase; PCK1/2, phosphoenolpyruvate carboxykinase 1/2; Glucose 6-P, glucose-6-phosphate; PEP, phosphoenolpyruvate; α-KG, α-ketoglutarate; Asp, aspartate; OAA, oxaloacetate.

We next characterized the expression profiles of genes involved in glucose and glutamine metabolism by quantitative real-time PCR (qRT-PCR). The control LKO C4-2B cells exhibited significantly higher expression levels of essentially all glycolysis genes (*HK2*, *PFK*, *ALDOA*, *GAPDH*, *PGM1*, *ENO1*, and* LDHA*) than those in knockdown cells (**Figure 3A**). Glucose transporter 1 (*GLUT1*) levels were lower in KDM4B-knockdown cells than in control cells, although the difference was not significant, indicating a reduced level of glucose uptake and glycolytic flux. The effects of knockdown on TCA cycle-related genes varied. The expression levels of *IDH2*, *SDHA*, and* MDH1* were reduced while levels of *SUCLG1* were elevated*.* qRT-PCR analysis further revealed that mitochondrial glutaminase 1 (*GLS1*), involved in tumor growth 44, and phosphoenolpyruvate carboxykinase (*PCK2*), which regulates tumor-initiating cells 45, were substantially downregulated by the depletion of KDM4B (**Figure 3A**). Consistent with the reduced expression of *GLS1*, there was a significant increase in glutamine but a lower degree of glutamate, as determined by LC-MS (**Figure 3B-F**). A significantly lower level of citrate in KDM4B-knockdown cells than in control cells was also found, supporting a reduced level of glutamine utilization.

Genes encoding pyruvate dehydrogenase kinases (*PDK1*, *PDK2*, and *PDK3*) governing the flux from pyruvate to mitochondria via the inhibition of PDH activity were also significantly downregulated in KDM4B-knockdown cells (**Figure 3A**). **Figure 3G** shows that the depletion of KDM4B indeed led to a significantly higher degree of PDH activity, indicating a higher flux from pyruvate to the TCA cycle and a potentially higher degree of OXOPHOS 46.

We then quantitatively analyzed glutathione and oxidized glutathione by LC-MS and evaluated ROS production using the H2DCFDA probe. As shown in **Figure 3E-F**, the level of glutathione was significantly reduced while oxidized glutathione increased in KDM4B-knockdown cells. Furthermore, the depletion of KDM4B significantly elevated the production of cellular ROS (*p* < 0.0001) (**Figure 3H**). These results suggest that the silencing of KDM4B greatly reduced glucose uptake, glycolytic flux, and glutamine usage but increased OXOPHOS metabolism, as depicted in **Figure 3I**.

### KDM4B interacts with c-Myc to modulate c-Myc-mediated transactivation

**Figure 4:**
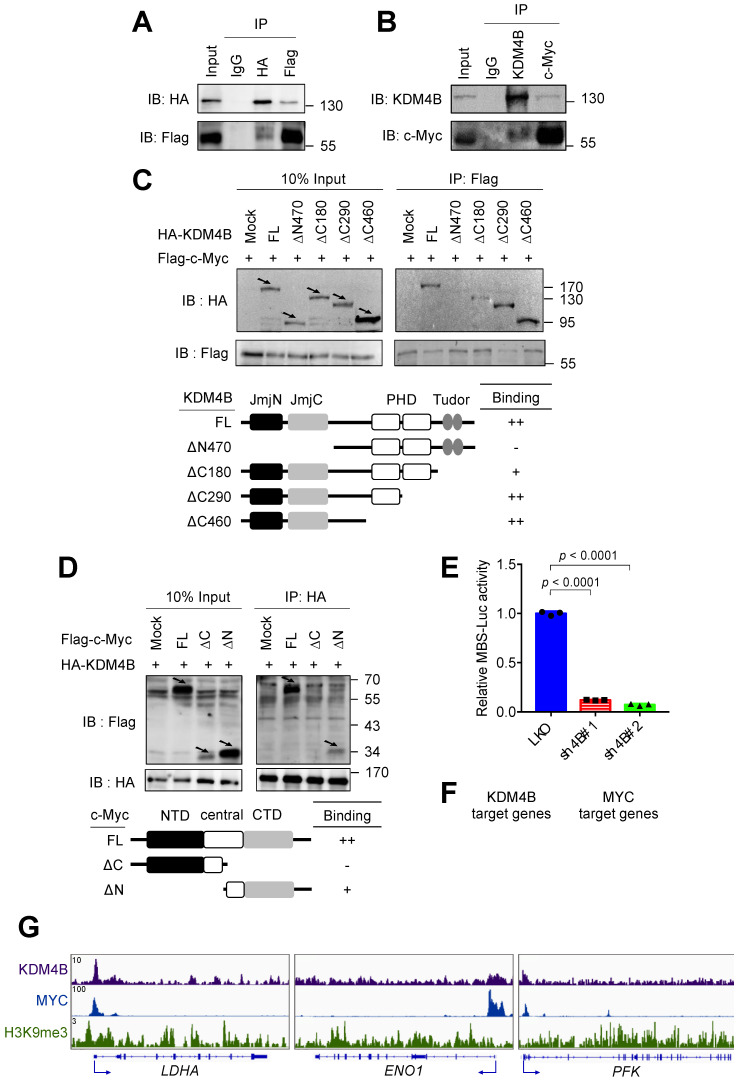
**KDM4B interacts with c-Myc. (A)** Association of ectopically expressed KDM4B and c-Myc. HEK293 cells were co-transfected with pCMV-HA-KDM4B and pCMV-flag-c-Myc as indicated. Immunoprecipitation (IP) was conducted using anti-HA or anti-Flag antibody, followed by immunoblotting analysis. Rabbit IgG was used as a negative control. **(B)** Endogenous association of KDM4B and c-Myc. C4-2B cell lysates were used for IP analysis with an anti-KDM4B or anti-c-Myc antibody as indicated, followed by immunostaining. Rabbit IgG was used as a negative control. **(C)** Co-IP assays were performed with an anti-Flag antibody in lysates from HEK293 cells co-transfected with a full-length (FL) Flag-c-Myc vector plus one of the HA-tagged constructs [mock, full length (FL) KDM4B, or KDM4B truncated mutants (∆N470, ∆C180, ∆C290, and ∆C460)], followed by immunoblotting analysis. Arrows indicate the signals of the full-length or the truncated proteins. **(D)** Co-IP assays were performed with an anti-HA antibody in lysates from HEK293 cells co-transfected with a full-length (FL) HA-KDM4B vector plus one of the Flag-tagged constructs [mock, full length (FL) c-Myc, or c-Myc truncated mutants (∆C and ∆N)], followed by immunoblotting analysis. NTD, N terminal domain; CTD, C terminal domain. Arrows indicate the signals of the full-length or the truncated proteins. **(E)** Regulation of c-Myc transactivation activity by KDM4B. C4-2B cells (LKO, sh4B#1, or sh4B#2) co-transfected with MBS-Luc plus β-galactosidase (an internal control). The activity was detected using Beta-Glo/One-Glo Assay System. **(F)** Overlap between KDM4B-bound (purple circle) and MYC-bound (blue circle) targets in K562 (myelogenous leukemia cell line) ChIP-seq datasets (https://www.encodeproject.org) using Venn diagram. **(G)** The binding peaks (grey) of KDM4B, MYC, and H3K9me3 at the proximal promoter region of *LDHA*, *ENO1*, and *PFK* in K562 (myelogenous leukemia cell line) ChIP-seq datasets. Statistical analysis was assessed by Student's *t*-test. Data are presented as the mean ± SD from three independent experiments.

c-Myc drives the expression of many metabolism-related genes, including *GLUT1*, *HK2*, *PKM2*, *LDHA*, and *GLS1* 12. We reasoned that KDM4B might function as a co-activator of c-Myc to regulate the expression of energy-related genes. We asked whether KDM4B physically interacts with c-Myc. An immunoprecipitation (IP) analysis of lysates from cells (293T) co-expressing HA-tagged KDM4B and Flag-tagged c-Myc revealed that KDM4B interacts with c-Myc (**Figure 4A**). This association was confirmed by an endogenous IP analysis of lysates from C4-2B cells (**Figure 4B**). We further mapped the interaction region between KDM4B and c-Myc using various HA-tagged forms of KDM4B; a full-length KDM4B (KDM4B-FL) as well as N-terminal and C-terminal (KDM4B-∆N470, KDM4B-∆C180, KDM4B-∆C290, and KDM4B-∆C460) truncation mutants fused to HA-tag were generated (**Figure 4C**, left panel). HEK293 cells were co-transfected with Flag-c-Myc and an HA vector or a variant of HA-KDM4B. As shown in **Figure 4C**, an IP analysis showed clear anti-HA signals for the full-length (FL) vector and each of three C-terminal mutants, whereas hardly any signal was detected for the *N*-terminal mutant (∆N470), indicating that the *N*-terminal region of KDM4B spanning the JmjN and JmjC region was crucial for binding to c-Myc. The reciprocal experiment was conducted for c-Myc by co-transfecting HEK293 with HA-KDM4B and a c-Myc expression vector with a Flag tag (Flag vector, c-Myc-FL, c-Myc-∆N, or c-Myc-∆C). An IP analysis revealed a clear binding signal for full-length c-Myc while much weaker affinity for c-Myc-∆C (**Figure 4D**), indicating that the C-terminal domain was important for binding to KDM4B.

To evaluate whether KDM4B regulates c-Myc-mediated transactivation activity, we used a c-Myc reporter plasmid consisting of 4 repetitive c-Myc binding sequences fused to a luciferase gene (MBS-Luc). Remarkably, the depletion of KDM4B essentially blocked transactivation activity in two independent knockdown lines, supporting the role of KDM4B as a c-Myc coactivator (**Figure 4E**). Consistently, genomic-wide analysis of KDM4B- and c-Myc-bound targets using the ChIP-seq data of K562 at ENCODE (https://www.encodeproject.org) 47 revealed that 72.6% (4134/5691) of KDM4B-bound targets also recruited c-Myc (**Figure 4F**). We examined the ChIP-seq peaks at the proximal promoter region of *LDHA*, *ENO1*, and *PFK*. **Figure 4G** shows a strong overlapped peak for KDM4B and c-Myc, respectively, whereas a much-reduced signal of H3K9me3 at the corresponding region, suggesting that KDM4B and c-Myc were co-recruited to those loci for a likely transactivation activity.

The expression of c-Myc-regulated genes including *LDHA*, *ENO1*, and *PFK* in tumor C4-2B grafts (LKO *vs.* sh4B#1) was then compared using qRT-PCR analysis*.* **Figure S7** shows the depletion of KDM4B in xenografts (**Figure S7A**) significantly reduced the expression of *LDHA*, *ENO1*, and *PFK*, were significantly decreased in KDM4B-knockdown (sh4B#1) cells (**Figure S7B-D**), consistent with the cell-based results (**Figure 3**). We next evaluated the effect of c-Myc silencing in C4-2B and CWR22Rv1. **Figure S8 s**hows that the depletion of c-Myc by siRNA (siMYC) led to a significantly lower expression of target genes (*LDHA*, *ENO1*, and *PFK*) as compared with scramble siRNA (siCTR).

We next sought to evaluate whether KDM4B is the key member of the KDM4 family (KDM4A, KDM4B, and KDM4C) for controlling c-Myc to regulate cancer metabolism. To this end, each of the KDM4 members was individually depleted using the lentiviral system; two knockdown clones (#1 and #2) of KDM4A, KDM4B, and KDM4C *vs.* control (LKO) in C4-2B and CWR22Rv1 were then evaluated. As shown in **Figure S9**, the depletion of KDM4B (sh4B#1 and sh4B#2) significantly decreased the level of *LDHA*, *ENO1*, and *PFK*. By contrast, knockdown of KDM4A or KDM4C did not individually reduced the expression of *LDHA*, *ENO1*, and *PFK*. Together, our results suggest the regulation of target genes by KDM4B and c-Myc.

We next asked whether the c-Myc-KDM4B interaction is important for cell proliferation. To do so, we compared the degree of cell proliferation in control (LKO) or KDM4B-knockdown (sh4B#1 or sh4B#2) C4-2B ectopically expressed with a control (mock) or c-Myc. **Figure S10A** shows a relatively comparable levels of c-Myc in the KDM4B knockdown as compared with the LKO. LKO ectopically expressed with c-Myc (LKO-c-Myc) exhibited a significantly higher degree of cell proliferation than did LKO-mock (*p* = 0.0080) or sh4B ectopically expressed with c-Myc (sh4B-c-Myc) (*p* < 0.0001) (**Figure S10B,** left panel). No significant difference was detected between KDM4B-knockdown and KDM4B-knockdown ectopically expressed with c-Myc (sh4B-mock *vs.* sh4B-c-Myc). Likewise, ectopic expression of c-Myc in LKO CWR22Rv1 significantly increased the degree of cell proliferation (*p* = 0.0138), whereas no significant difference was detected in KDM4B-knockdown ectopically expressed with c-Myc as compared with KDM4B-knockdown (**Figure S10B**, right panel).

**Figure 5:**
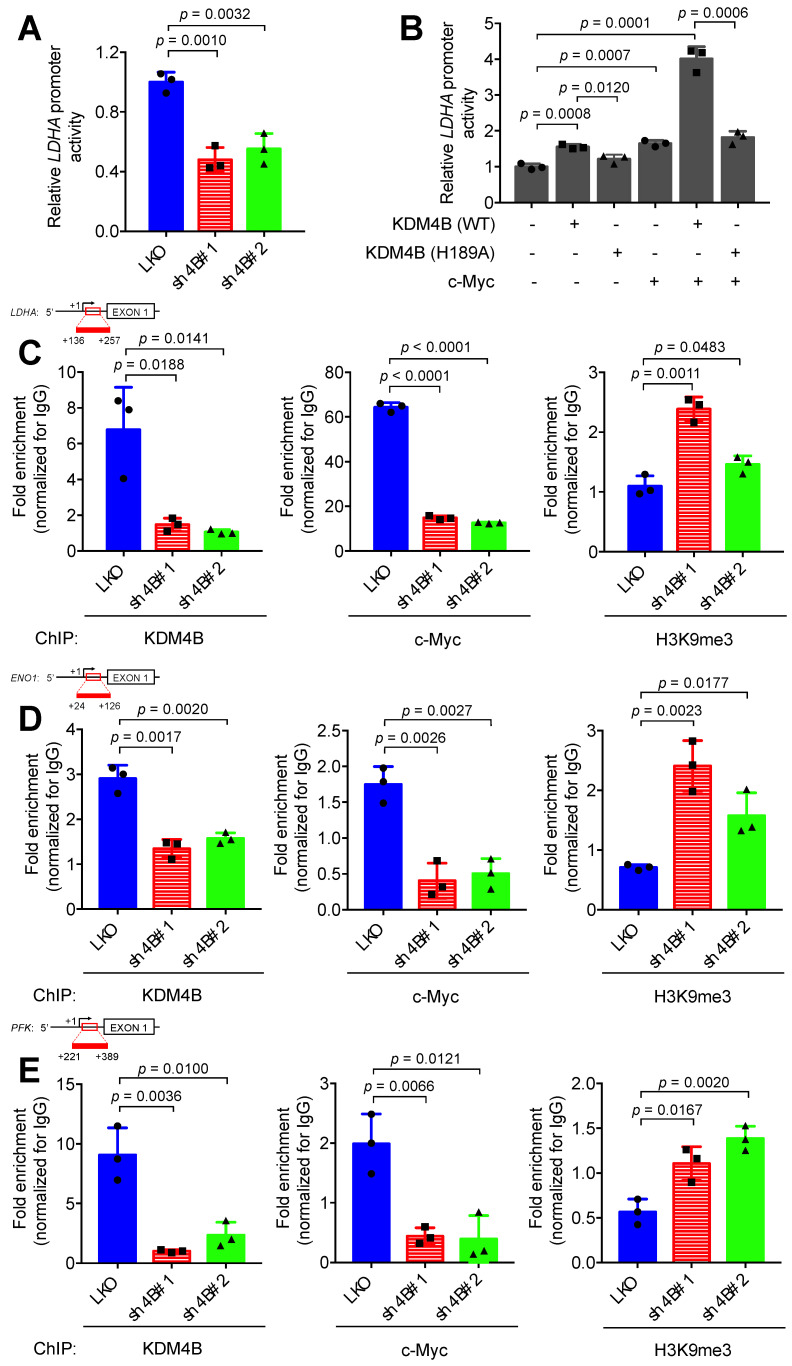
** KDM4B and c-Myc are co-recruited at the *LDHA* locus. (A-B)** Transactivation activity of C4-2B cells (LKO, sh4B#1, or sh4B#2) transfected with a *LDHA* reporter construct **(A)** or C4-2B co-transfected with a *LDHA* reporter construct, a KDM4B vector [KDM4B wild-type (WT), inactive KDM4B mutant (H189A)], and/or c-Myc. (**B**) The Beta-Glo/One-Glo Assay System was applied to assess the luciferase activity. β-galactosidase was an internal control. **(C-E)** Chromatin immunoprecipitation (ChIP) analysis of KDM4B and c-Myc occupancy on the* LDHA* **(C)**,* ENO1* **(D)**, and* PFK* **(E)** promoter loci. ChIP was performed with specific antibodies against KDM4B, c-Myc, and H3K9me3, respectively in control (LKO) and KDM4B-knockdown (sh4B#1 and sh4B#2) C4-2B cells. ChIP DNAs were analyzed by qPCR with primers that amplify genomic regions. Data were presented as the average of fold enrichment normalized to IgG ± SD from three independent experiments. Statistical analysis was assessed by Student's *t*-test.

To further confirm the notion that KDM4B is crucial for c-Myc-mediated metabolic change, we determined the glycolytic capacity in LKO or KDM4B-knockdown C4-2B ectopically expressed with mock or c-Myc using the Seahorse analyzer. **Figure S10C** shows that the degree of glycolytic capacity was significantly increased in LKO-c-Myc as compared with LKO-mock, sh4B-c-Myc, or sh4B-mock. Instead, sh4B-c-Myc did not show significantly increased glycolytic capacity as compared with sh4B-mock, possibly because of a scarce level of KDM4B in the KDM4B knockdown. By the use of CWR22Rv1, there were also comparable metabolic profiles (**Figure S10D**). Thus, it is likely that KDM4B functions as a critical coactivator of c-Myc to achieve its full transactivation activity. KDM4B is important for the chromatin relaxation of c-Myc target genes as evidenced in **Figure 5**. Without KDM4B, even with abundant c-Myc, full transactivation cannot be achieved. Collectively, our results suggest that KDM4B serves as a crucial co-activator of c-Myc engaged in metabolic regulation and cell proliferation.

### KDM4B is co-recruited with c-Myc to *LDHA*, *ENO1*, and *PFK* promoters

To confirm that KDM4B co-activates c-Myc transactivation, we used an *LDHA* promoter activity assay with a reporter construct consisting of an *LDHA* promoter region fused to luciferase. The results indicated that cells of C4-2B with KDM4B knockdown had significantly lower promoter activity levels than those in control cells (**Figure 5A**). The depletion of KDM4B in CWR22Rv1 also exhibited comparable results (**Figure S11A**). In addition, C4-2B ectopically expressed KDM4B (4B) exhibited a significantly higher level of promoter activity than did the control (mock) (4B *vs.* mock, *p* = 0.0065) (**Figure S11B**). Restoration of KDM4B in KDM4B-knockdown C4-2B also significantly rescued the promoter activity as compared to the control cells (sh4B#1-resi. *vs.* sh4B#1-mock, *p* = 0.0001; sh4B#2-resi. *vs.* sh4B#2-mock, *p* = 0.0003) (**Figure S11B**). There were also comparable results using the CWR22Rv1 model (**Figure S11C**).

We next asked whether the overexpression of KDM4B (WT) or c-Myc could elevate the transactivation activity and whether demethylase activity was necessary using a KDM4B dead mutant (H189A). The ectopic expression of KDM4B (WT) or c-Myc alone significantly increased *LDHA* promoter activity. This increase was abolished by the introduction of the dead KDM4B mutant. When cells were co-transfected with KDM4B (WT) and c-Myc, remarkably, there was a synergistic increase (4.0-fold), and this increase was attenuated in cells co-transfected with KDM4B (H189A) and c-Myc (**Figure 5B**). These results suggest that KDM4B co-activated c-Myc-mediated transactivation via demethylase activity.

We next assessed whether KDM4B and c-Myc were directly co-recruited to the *LDHA* locus using a ChIP analysis. We showed that KDM4B was recruited to the *LDHA* locus, and its depletion by shRNA reduced its occupancy in C4-2B cells (**Figure 5C**, left panel). Similarly, a ChIP analysis revealed that c-Myc was recruited to this locus, and its occupancy relied on the presence of KDM4B, as KDM4B knockdown significantly abolished this recruitment (**Figure 5C**, center panel). Consistent with these results, signals corresponding to the H3K9me3 mark on the *LDHA* locus were significantly higher in KDM4B-knockdown cells than in control cells (**Figure 5C**, right panel**)**. Using UCSC genome browser tool, we examined whether KDM4B and c-Myc were co-recruited to the promoter region of differentially expressing glycolysis-related genes in **Figure 3A**. Of those, the *ENO1* and *PFK* loci showed strong binding signals of KDM4B and c-Myc. This was further confirmed using ChIP analyses (KDM4B, c-Myc, and H3K9me3) in C4-2B (LKO *vs.* sh4B#1 and sh4B#2) (**Figure 5D-E**).

We also evaluated whether the demethylase activity of KDM4B was required for promoter binding. A ChIP analysis between KDM4B WT and H189A in C4-2B revealed that there was no significant difference in binding to the promoter loci of energy-related genes (*LDHA, ENO1*, and *PFK*) between WT and the inactive mutant (H189A) (**Figure S12B-D**), indicating that the enzymatic activity of KDM4B was not important for binding to the promoter region. Instead, the WT significantly removed the signal of H3K9me3 from energy-related genes (*LDHA, ENO1*, and *PFK*) as compared with H189A. The depletion of KDM4B or c-Myc led to a significantly lower expression of target genes as compared to the control cells in either C4-2B or CWR22Rv1 (**Figure 3A** and **Figure S8**). Collectively, our results suggest that KDM4B interacts with c-Myc and they together regulate c-Myc-mediated transactivation via H3K9me3/2 demethylase activity.

### KDM4B and c-Myc are co-localized in PCa and are significantly associated with worse survival

**Figure 6:**
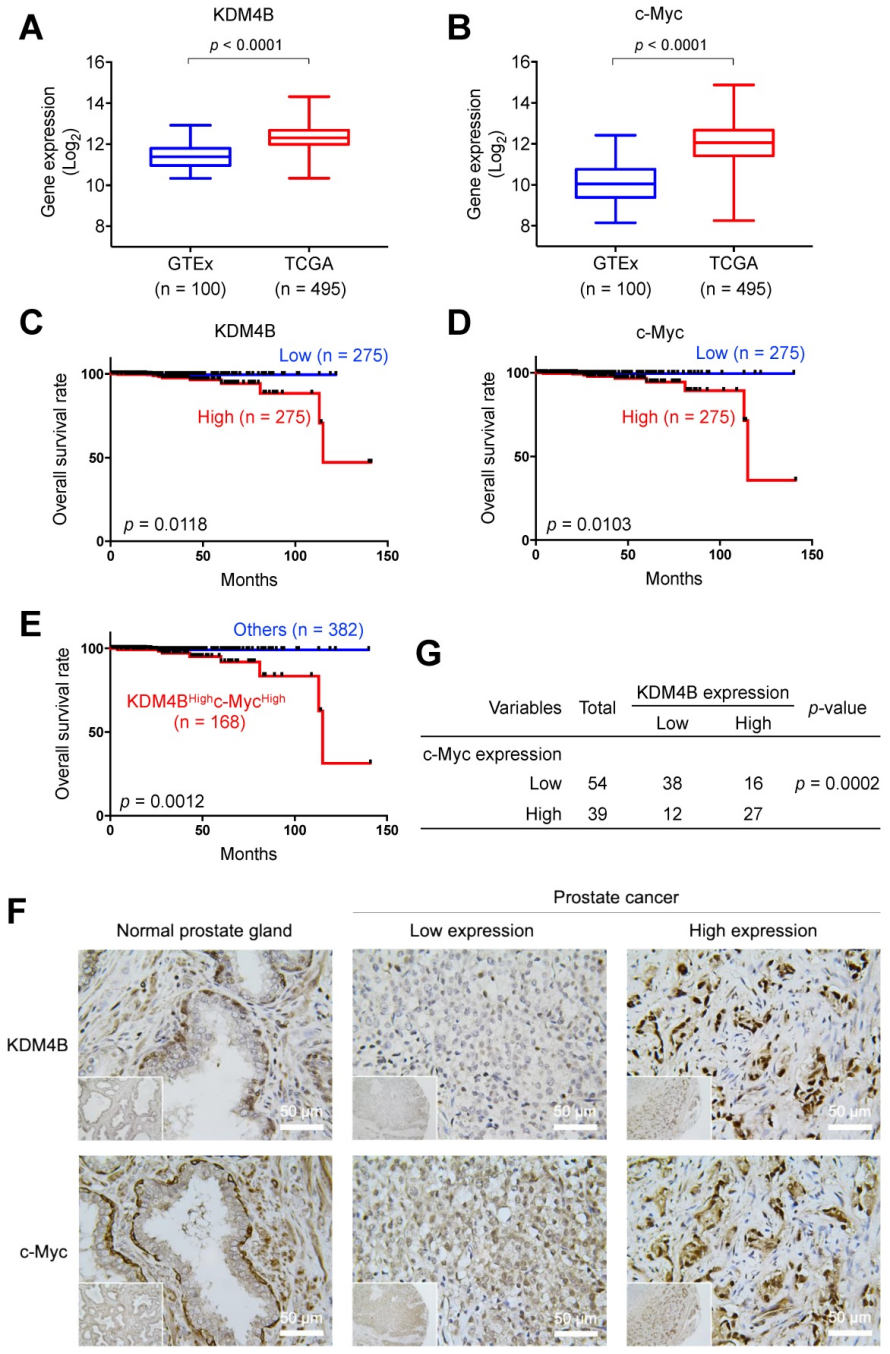
** KDM4B and c-Myc are co-overexpressed in PCa and are associated with a poor overall survival. (A-B)** The prostate tissue gene expression of KDM4B** (A)** and c-Myc **(B)** were analyzed in TCGA TARGET GTEx dataset from UCSC Xena (https://xenabrowser.net). **(C-E)** Elevated expression of KDM4B **(C)** or c-Myc **(D)** alone, or both **(E)** is significantly correlated with worse overall survival of PCa patients in TCGA PRAD dataset from UCSC Xena. The cutoff value of high and low expression was set as the median. **(F)** Representative images of PCa specimens (n = 93) stained with anti-KDM4B and anti-c-Myc, and separated based on the median value. Scale bar, 50 μm. **(G)** The correlation between KDM4B expression and c-Myc signal in 93 prostate biopsies from **(F)**. Statistical analysis was assessed by Chi-square test.

To evaluate the prognostic value of KDM4B and c-Myc in PCa, we surveyed the expression profile of KDM4B in normal prostatic tissues and prostate cancer tissues using UCSC Xena (https://xena.ucsc.edu) and publicly available genome datasets 48. As shown in **Figure 6A**, KDM4B expression was significantly higher in the PCa tumors (TCGA, n = 495) than in normal prostate tissues (GTEx, n = 100). Similarly, c-Myc expression was significantly higher in cancer specimens than in normal tissues (**Figure 6B**). Additionally, a Kaplan-Meier analysis using data of PCa patients extracted from UCSC Xena revealed that elevated KDM4B expression is significantly associated with a worse overall survival (OS) (*p* = 0.0118) (**Figure 6C**). High expression of c-Myc was also significantly associated with a poor OS (*p* = 0.0103) (**Figure 6D**). Importantly, the combined double-high status offered an even greater statistical significance (KDM4B^High^c-Myc^High^ *vs.* Others, *p* = 0.0012) (**Figure 6E**).

We next characterized the expression of KDM4B and c-Myc in consecutive sections of paraffin-embedded prostatic cancer specimens from a tissue microarray (n = 93) by immunohistochemistry. **Figure 6F** shows representative images of KDM4B and c-Myc expression patterns. IHC results were scored based on two parameters: the intensity grade (score: 1‒3) and proportion of positive tumor cells (score: 0‒100 based on a multiple of 5) in the nucleus 49. The immunoreactive score was obtained by multiplying the two parameters. Samples were stratified into high and low expression based on the median value. Strikingly, there is a significantly strong co-occurrence between KDM4B and c-Myc (*p* = 0.0002) (**Figure 6G**). These results together suggest that KDM4B and c-Myc were co-expressed in prostate cancer and were associated with poor outcomes in PCa.

## Discussion

KDM4B is a potential oncogenic demethylase with the ability to demethylate the repressive modifications H3K9me3/2 on the chromatin 23. In this study, we have found that KDM4B physically interacts with c-Myc, a key oncogenic transcription factor involved in the initiation and progression of PCa 12, 15, 50. Notably, the depletion of KDM4B substantially reduced the expression of important metabolism-related genes mediated by c-Myc (*GLUT1*, *HK2*, *PFK*, *ENO1*, *PKM2*, *LDHA*, and *GLS1*), suggesting that KDM4B functions as a co-activator of c-Myc. Our ChIP analyses of the *LDHA*,* PFK*, and* ENO1* loci further showed that KDM4B and c-Myc are co-recruited to the promoter region of *LDHA*,* PFK*, and* ENO1.*

An earlier report from Yang *et al.* reveals that KDM4B physically interacts with N-Myc, a Myc member that shares ~36% sequence identity with c-Myc 51. Interestingly, the KDM4B-N-Myc complex transactivates a specific subset of genes involved in neuroblastoma progression 51. Additionally, Yang *et al.* elegantly provides the mechanistic basis of chromatin remodeling process by this complex, where N-Myc first binds DNA and then recruits KDM4B to remove the surrounding H3K9me3/2 marks so as to unwrap the chromatin. Thus, it is speculated that c-Myc might follow a similar strategy to cooperate with KDM4B for a full transactivation activity. The strikingly synergistic effect seen in the demethylase-dependent* LDHA* promoter activity greatly supports this notion. Taken together, KDM4B serves as an important epigenetic regulator to cooperate with c-Myc/N-Myc in chromatin relaxation and transactivation, in accord with our previous work 40.

We show that the depletion of KDM4B leads to a metabolic switch from aerobic glycolysis to OXPHOS; the levels of ECAR, glutathione, and glutamate were reduced, while OCR, oxidized glutathione, glutamine, and ROS levels were significantly elevated. While overexpressing KDM4B reversed this phenotype, overexpression of c-Myc failed to do so. This suggests that KDM4B knockdown not only decreased the level of c-Myc expression (see below) but also its transcriptional activity, which cannot be compensated by mere overexpression. KDM4B, by serving as a coactivator of c-Myc relaxes the chromatin near c-Myc binding sites (**Fig. 5**) and plays an important role in c-Myc's transactivation potential. Thus, KDM4B enhances c-Myc's activity in AR positive PCa cells by at least two ways, 1) coactivating AR to drive c-Myc expression, and 2) coactivating c-Myc to drive the expression of target genes. Together, KDM4B in cooperation with c-Myc is essential to promote Warburg metabolism, and at the same time, to prevent mitochondrial overheating by suppressing excessive formation of ROS.

Consistent with KDM4B being a Warburg switch, we found its depletion of KDM4B in C4-2B significantly increased PDH activity to augment mitochondrial functions. We asked whether suppressing PDH activity by the depletion of pyruvate dehydrogenase A1 (PDHA1) using siPDHA1 would return KDM4B-deprived cells to glycolytic state and restore the proliferation of the cells. We did not see restoration of cell growth by siPDHA1 in shKDM4B knockdown cells (**Figure S13**). In fact, siPDHA1-treated control LKO cells also showed significantly reduced level of cell proliferation, indicating PDHA1 is critical for the growth of CWR22Rv1, in agreement with previously reported data that the depletion of PDHA1 suppresses the growth of CWR22Rv1 52. Further investigation is needed to evaluate the metabolic dependence in the context of the KDM4B status, tumor genetic background, or the composition of the tumor microenvironment.

KDM4B is a transcriptional activator of AR 30. Of note, AR regulates the expression of c-Myc in PCa 52. Furthermore, KDM4B is induced by p53 and acts in an auto-regulatory loop to moderate the p53 response in response to DNA damage 53. We previously showed that the inhibition of KDM4B effectively suppresses the proliferation of LNCaP, an androgen-sensitive PCa, using a lentivirus-based knockdown approach and a small-molecule inhibitor 31. Duan *et al.* has shown that small KDM4 inhibitors greatly suppressed cell viability in PCa cells *in vivo* 36*.* Here, we further demonstrated that the depletion of KDM4B by specific shRNAs greatly curbed the proliferation of two CRPC cell lines *in vitro* and in xenografts. The potent inhibitory effects may be attributed to KDM4B being a dual co-activator of both AR and c-Myc. AR and c-Myc are both drivers of prostate cancers. Previous work nicely showed that KDM4B, being a coactivator of AR 30, drives c-Myc expression through recruitment of AR to the* Myc* gene enhancer in endocrine therapy resistant models of PCa 38. Our study further revealed that KDM4B is associated with c-Myc and serves as a coactivator of c-Myc. As such, it directly enhances Myc's transcriptional activity and up-regulates the expression of the energy-related genes involved in metabolism, promoting CRPC progression. Of note, p53-induced upregulation of KDM4B might additionally drive c-Myc expression in response to DNA damage 53. It is likely that the observed effects in KDM4B seen here are the combined results of c-Myc expression level and its activity. Thus, two modes of action synergize with each other and makes KDM4B a particularly suitable target for AR and c-Myc-driven tumors. The reduced recruitment of c-Myc to metabolic gene promoters in KDM4B depleted cells is likely due to the lower expression level of c-Myc as well as the more compact, H3K9me3-enriched chromatin environment. We also provided evidence that KDM4B and c-Myc are co-expressed in PCa tissue and that high expression of both is associated with poor clinical outcome. Our results thus provide proof-of-concept evidence that KDM4B inhibitor can be beneficially used to target both AR and c-Myc driven prostate cancers.

It is interesting that both KDM4A and KDM4B are overexpressed in PCa. Although KDM4A and KDM4B share a similar domain organization and a conserved active-site framework, the sequence identity in the DNA-binding region is relatively low (~50% identity) 31. Furthermore, KDM4A and KDM4C function as a homodimer or a heterodimer through the JmjN domain for their demethylase activity, whereas KDM4B functions as a monomer and does not heterodimerize with KDM4A or KDM4C 54. Given these features, it is likely that KDM4A and KDM4B display somewhat overlapping, but distinct spatiotemporal expression profiles, ensuring that they function combinatorically and independently to control non-redundant subsets of gene targets 55, 56. Thus, KDM4A and 4B are expected to have their unique chromatin binding sites, but also similar ones. In support of this notion, our earlier studies have shown that the KDM4A-E2F1 axis promotes tumor growth and metabolic adaptation through the regulation of the expression of cell-cycle related genes and *PDK1*/*PDK3* 32. The results of this study also revealed that KDM4B regulates the expression of *PDK*s. Of note, the interaction region of KDM4A with E2F1 is within the JmjC domain and its adjacent region (249-600 residues), a region with high conservation with KDM4B (84%), implicating that KDM4B might regulate *PDK*s via E2F1. Further studies are required to clarify how KDM4A and KDM4B coordinately contribute to metabolic adaptation.

**Figure 7:**
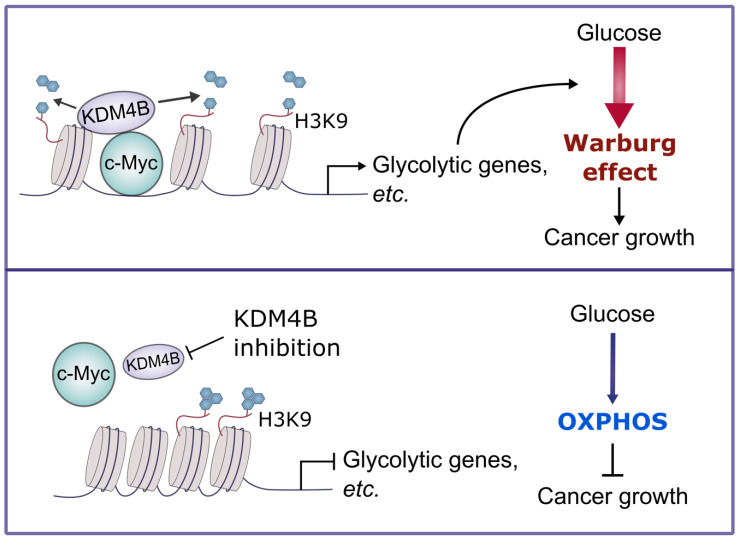
**A schematic diagram depicts the mechanism of the KDM4B-c-Myc complex in prostate cancer.** KDM4B interacts with c-Myc and regulates the metabolic genes including glycolytic genes via a demethylase-dependent manner, promoting the Warburg effect to accelerate the prostate cancer growth. Inhibition of KDM4B expression by specific shRNAs or small molecule inhibitors switches to an OXPHOS metabolism and curbs tumor growth.

In sum, we demonstrate that KDM4B acts as a co-activator of c-Myc, a widely oncogenic factor implicated in tumor development and progression. We further provide evidence that KDM4B and c-Myc work together to modulate energy metabolism in CRPC, and the inhibition of KDM4B decreases tumor growth via a metabolic switch (**Figure 7**). Accordingly, KDM4B is an ideal candidate target for advanced prostate cancer given its dual function to coactive c-Myc and AR.

## Supplementary Material

Supplementary figures and table.

## Abbreviations

AR: androgen receptor
ChIP: chromatin immunoprecipitation
CRPC: castration-resistant prostate cancer
ECAR: extracellular acidification rate
FL: full length
IP: immunoprecipitation
OCR: oxygen consumption
OXPHOS: oxidative phosphorylation
PCa: prostate cancer
qRT-PCR: quantitative real-time PCR
ROS: reactive oxygen species
sh4B: KDM4B knockdown
TCA cycle: tricarboxylic acid cycle
WT: wild-type
