# Supplementary material for: Targeting KDM4B that coactivates c-Myc-regulated metabolism to suppress tumor growth in castration-resistant prostate cancer
Source: Theranostics. 2021 Jun 26;11(16):7779–96. doi: 10.7150/thno.58729 (PMC8315051; doi:10.7150/thno.58729)
Supplement: Supplementary file 1 — Supplementary figures and table. [file thnov11p7779s1.pdf]

Supplementary figures

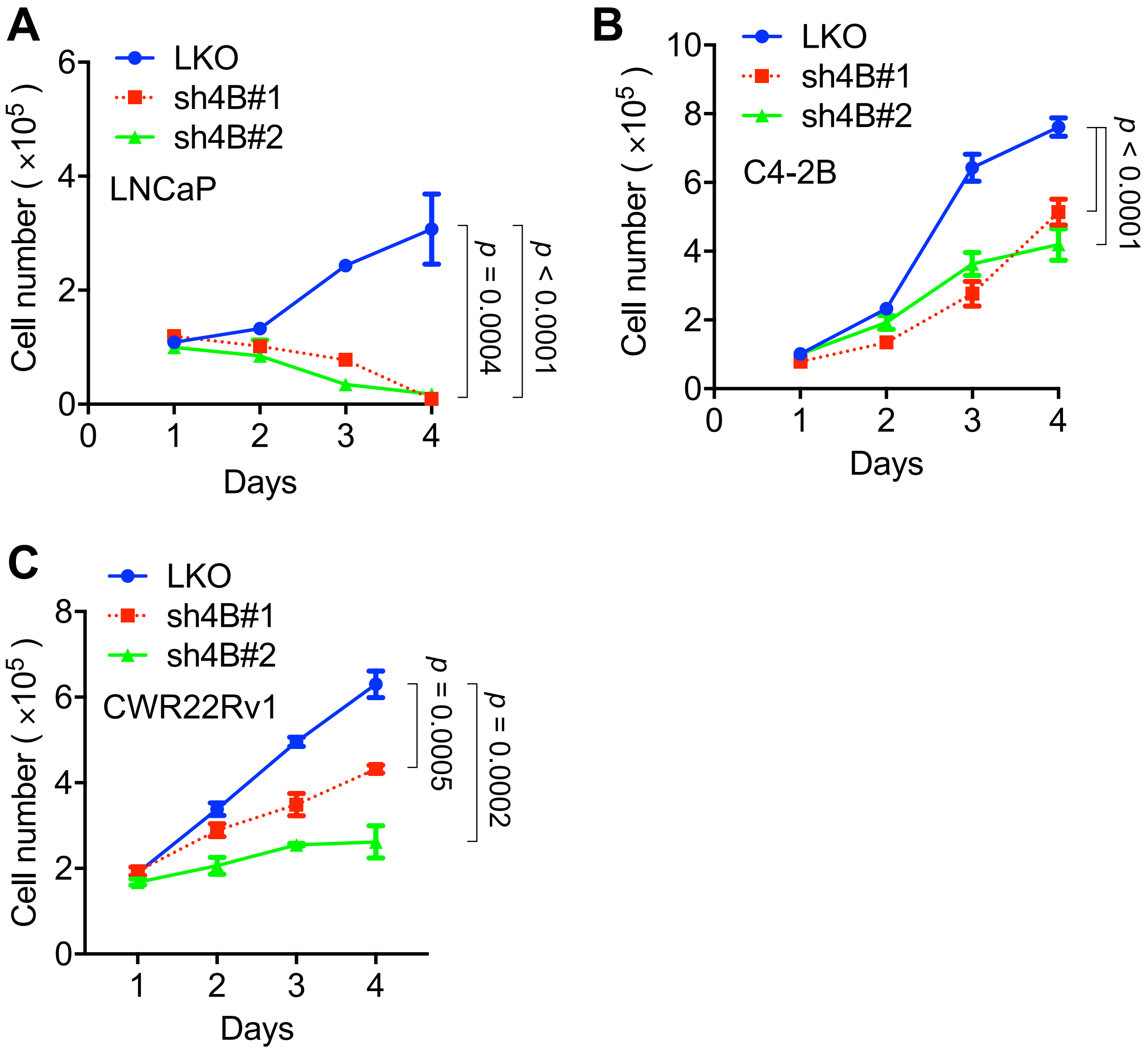

Figure S1. Inhibition of KDM4B reduces cell growth in AR-positive PCa cells. (A–C) Cell proliferation of control (LKO) and KDM4B-knockdown (sh4B#1 and sh4B#2) LNCaP **(A)**, C4-2B **(B)**, and CWR22Rv1 **(C)** was determined by cell count at indicated time points. Data presented are means ± SD from three independent experiments. Statistical analysis of the differences between two groups was calculated by two-sided Student's *t*-test.

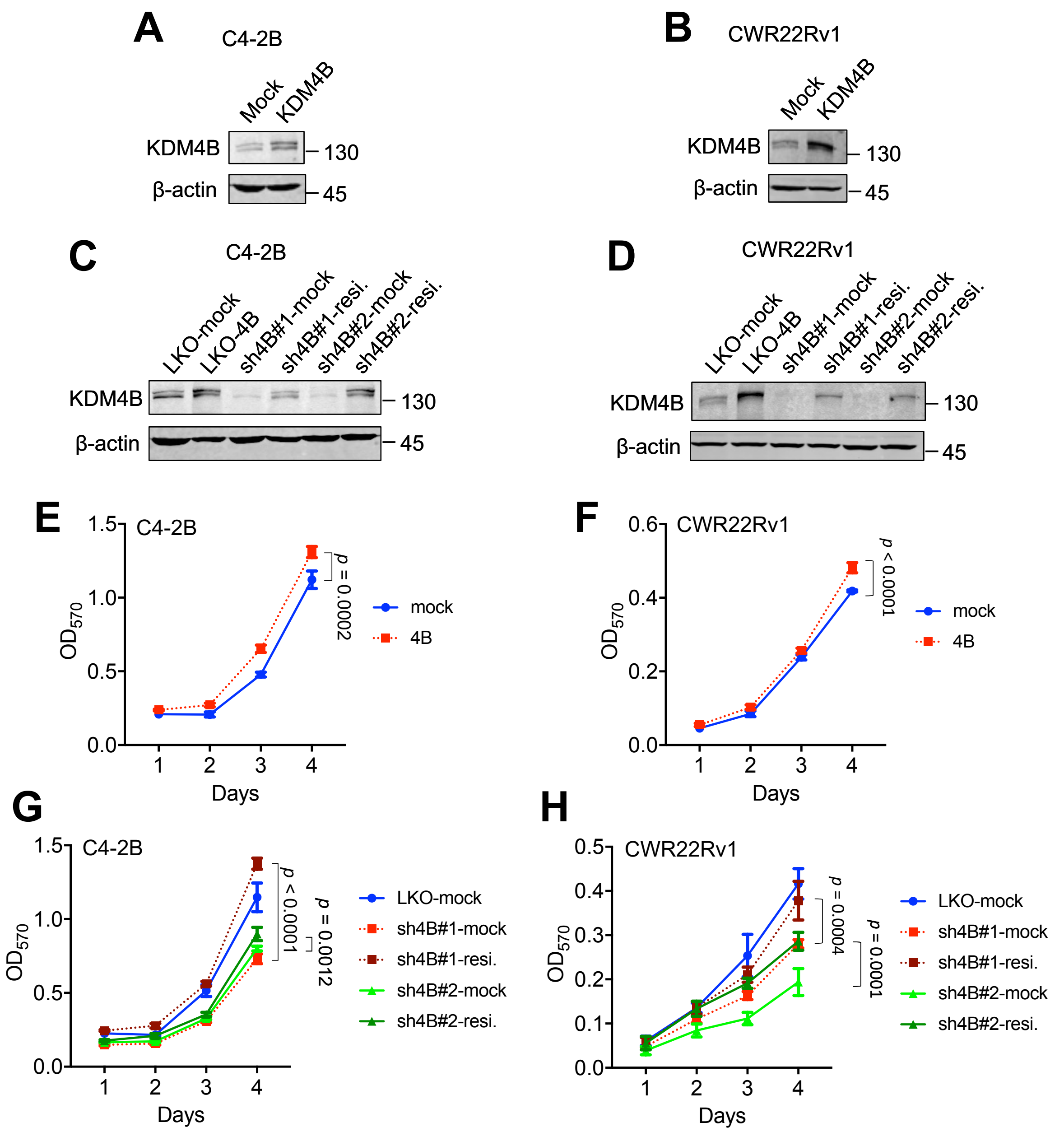

Figure S2. KDM4B is crucial for CRPC tumor growth. (**A–B**) C4-2B (**A**) or CWR22Rv1 (**B**) cells were transfected with a mock or KDM4B expression vector as indicated, followed by immunoblotting analysis. (**C–D**) C4-2B (**C**) or CWR22Rv1 (**D**) cells (LKO, sh4B#1, sh4B#2) were transfected with a mock or a specific shRNA-resistant KDM4B vector as indicated, followed by immunoblotting analysis. β-Actin was the internal control. (**E–F**) MTT cell proliferation assay of control (mock) and KDM4B overexpression (4B) in C4-2B (**E**) or CWR22Rv1 (**F**) at indicated time points. (**G–H**) MTT cell proliferation assay of KDM4B restoration in C4-2B (**G**) or CWR22Rv1 (**H**). Cells (LKO, sh4B#1, or sh4B#2) were transfected with an empty vector (mock), or a specific shRNA-resistant KDM4B vector (resi.). Cell proliferation was detected using MTT assay. Data are presented as means ± SD from three independent experiments. Statistical analysis of the differences between two groups was calculated by two-sided Student's *t*-test.

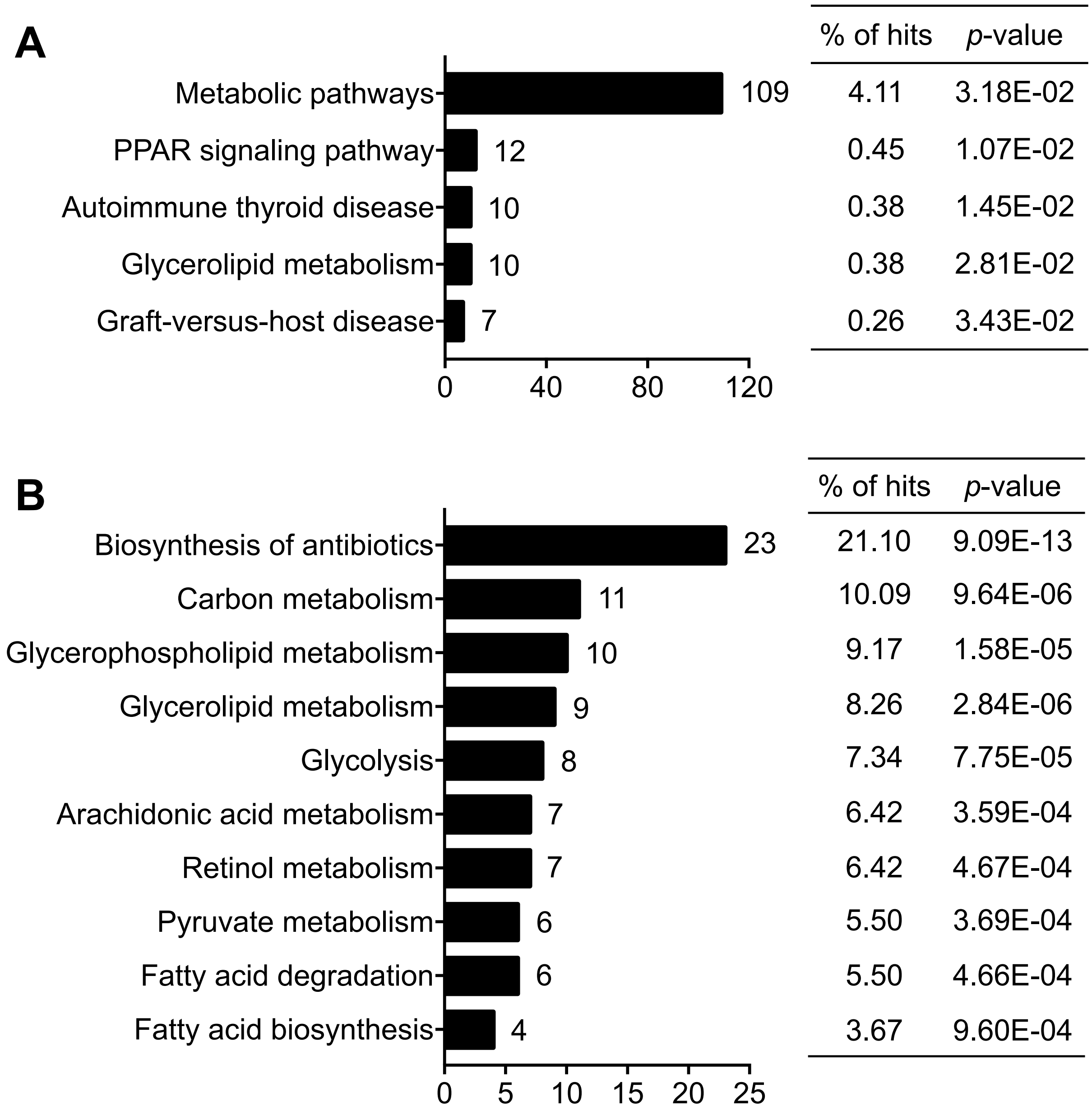

Figure S3. KDM4B regulates the expression of genes involved in metabolic pathways. (A) Top-ranking 3000 down-regulated genes (Log2 (fold change) < -1.25) in KDM4B knockdown C4-2B cells as compared to the control LKO cells that obtained from microarray (GEO number: GSE147481) were analyzed by KEGG pathway using DAVID (https://david.ncifcrf.gov). **(B)** The genes in metabolic pathways (n = 109) from **(A)** were analyzed by KEGG pathway using DAVID.

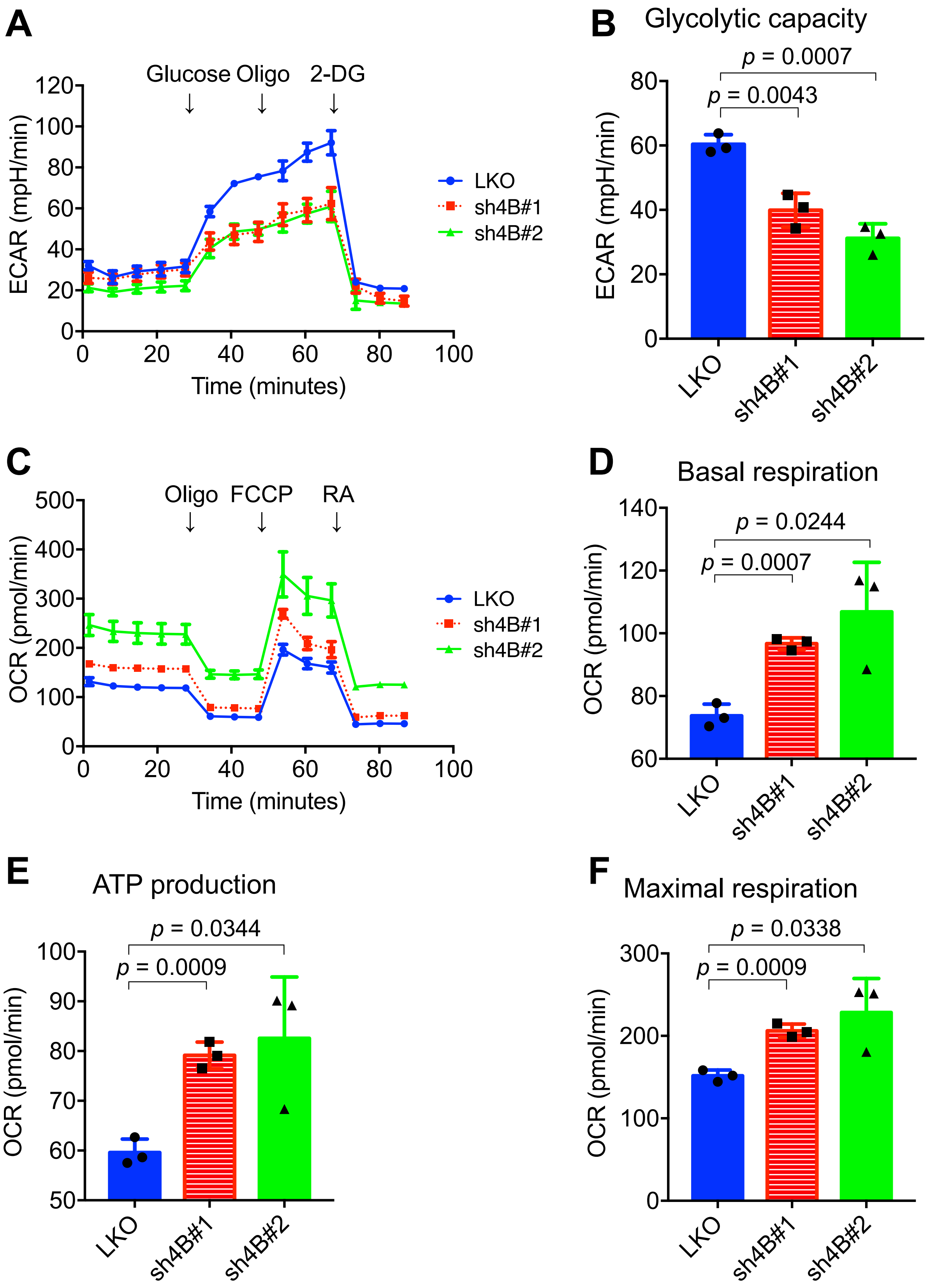

Figure S4. Depletion of KDM4B alters the EACR and OCR in CWR22Rv1 cells. (**A** and **C**) The extracellular ECAR **(A)** and OCR **(C)** of KDM4B-knockdown (sh4B#1 and sh4B#2) and control (LKO) CWR22Rv1 cells were measured by seahorse analyzer. For ECAR, adherent cells were incubated at glucose-free and $NaHCO_3$-free medium with subsequent injection of 10 mM glucose, 1 µM oligomycin

(Oligo), and 50 mM 2-DG. For OCR, adherent cells were incubated at $NaHCO_3$-free medium with subsequent injection of 1 μM oligomycin (Oligo), 1 μM FCCP, and 50 μM rotenone/antimycin A (RA). (**B**) The glycolytic capacity was plotted from (**A**). (**D–F**) The contribution of associated parameters of (**C**) including basal respiration (**D**), ATP production (**E**), and maximal respiration (**F**). All data are presented as the mean ± S.D. and p values were calculated by two-sided Student's t-test.

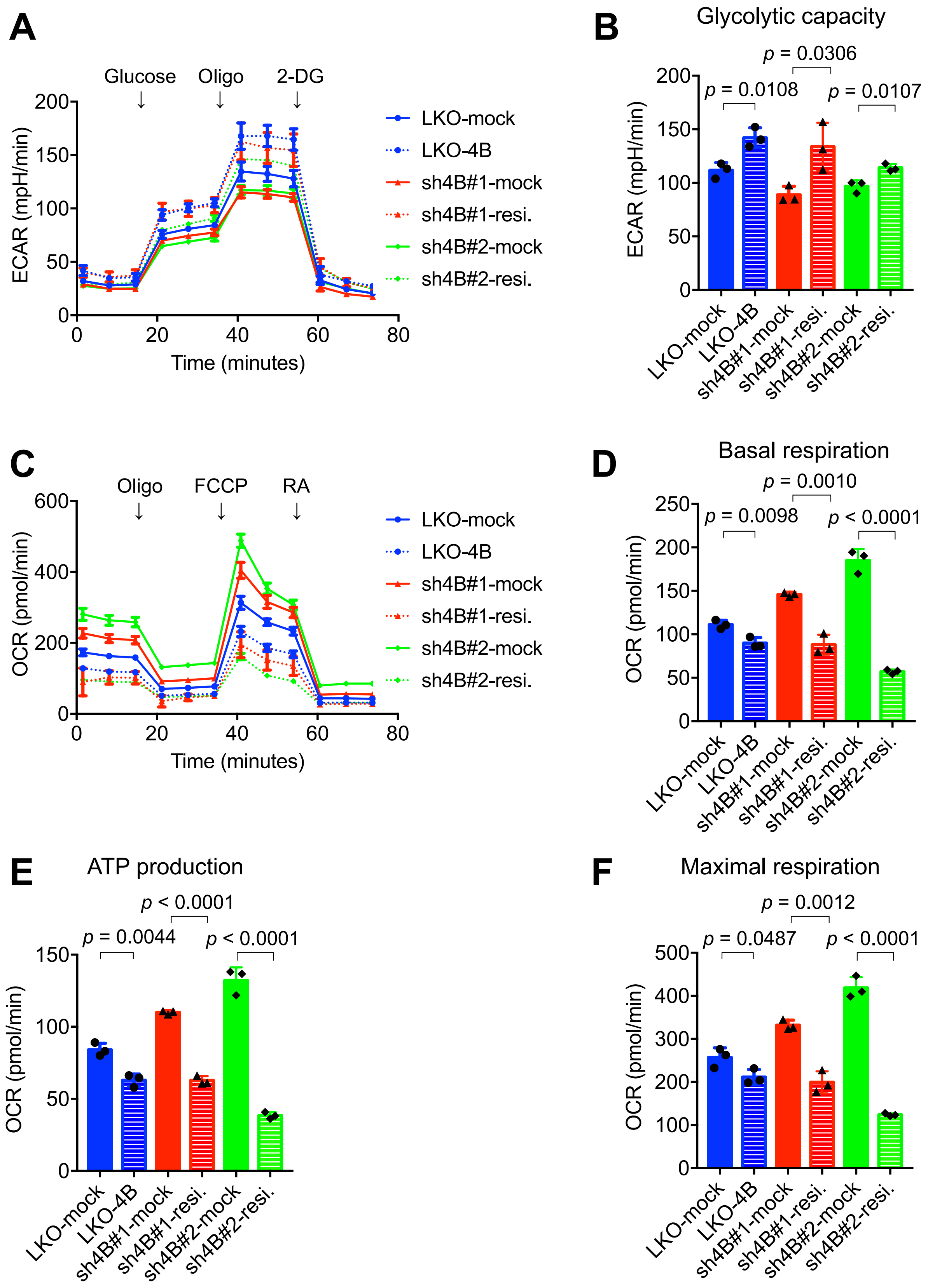

Figure S5. Restoration of KDM4B alters the EACR and OCR in C4-2B cells. (**A** and **C**) The extracellular ECAR **(A)** and OCR **(C)** of C4-2B cells were measured by seahorse analyzer. Cells (LKO, sh4B#1, and sh4B#2) were transfected with empty vector (mock), KDM4B expression vector (4B), or specific shRNA-resistant KDM4B expression vector (resi.). For ECAR, adherent cells were incubated at

glucose-free and $NaHCO_3$-free medium with subsequent injection of 10 mM glucose, 1 μM oligomycin (Oligo), and 50 mM 2-DG. For OCR, adherent cells were incubated at $NaHCO_3$-free medium with subsequent injection of 1 μM oligomycin (Oligo), 0.5 μM FCCP, and 50 μM rotenone/antimycin A (RA). (**B**) The glycolytic capacity was plotted from (**A**). (**D–F**) The contribution of associated parameters of (**C**) including basal respiration (**D**), ATP production (**E**), and maximal respiration (**F**). All data are presented as the mean ± S.D. and *p* values were calculated by two-sided Student's *t*-test.

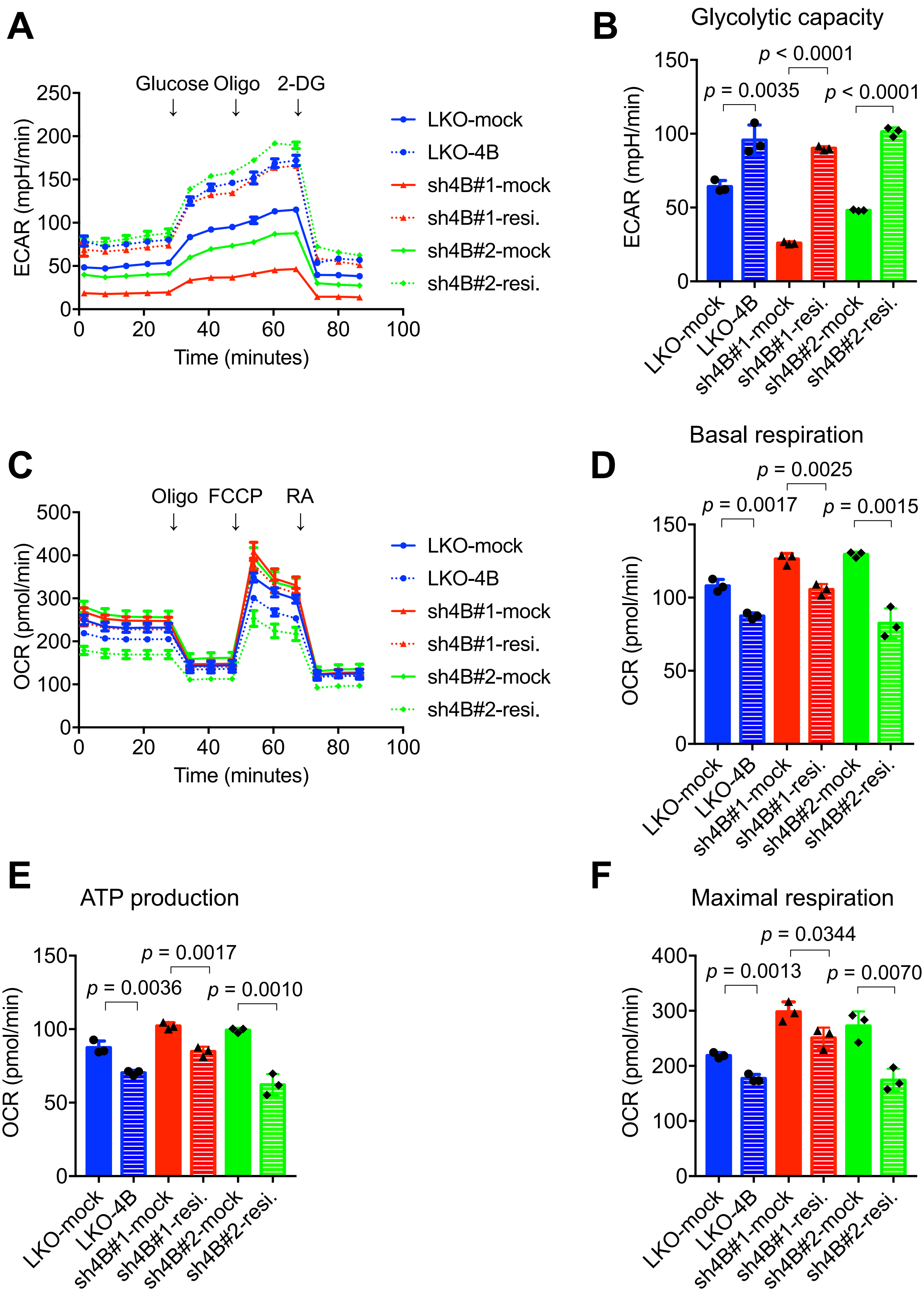

Figure S6. Restoration of KDM4B alters the EACR and OCR in CWR22Rv1 cells. (**A** and **C**) The extracellular ECAR **(A)** and OCR **(C)** of CWR22Rv1 cells were measured by seahorse analyzer. Cells (LKO, sh4B#1, and sh4B#2) were transfected with empty vector (mock), KDM4B expression vector (4B), or specific shRNA-resistant KDM4B expression vector (resi.). For ECAR, adherent cells were

incubated at glucose-free and $NaHCO_3$-free medium with subsequent injection of 10 mM glucose, 1 μM oligomycin (Oligo), and 50 mM 2-DG. For OCR, adherent cells were incubated at $NaHCO_3$-free medium with subsequent injection of 1 μM oligomycin (Oligo), 1 μM FCCP, and 50 μM rotenone/antimycin A (RA). (**B**) The glycolytic capacity was plotted from (**A**). (**D–F**) The contribution of associated parameters of (**C**) including basal respiration (**D**), ATP production (**E**), and maximal respiration (**F**). All data are presented as the mean ± S.D. and *p* values were calculated by two-sided Student's *t*-test.

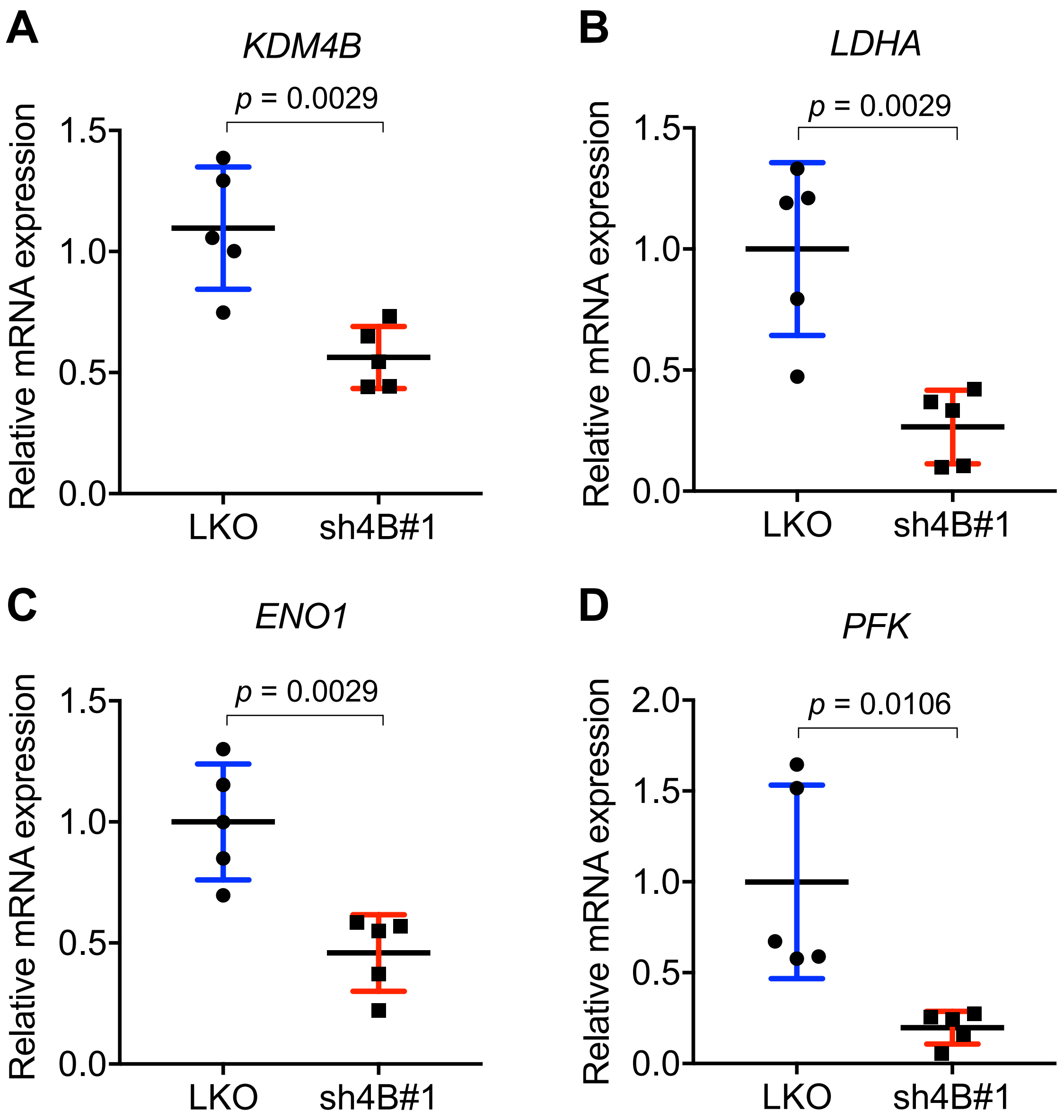

Figure S7. Inhibition of KDM4B downregulates the expression of glycolysis genes in the xenografts. (A–D) The relative mRNA expression levels of *KDM4B* **(A)**, *LDHA* **(B)***, ENO1* **(C)**, and *PFK* **(D)** in the LKO or KDM4B-knockdown (sh4B#1) xenografts. All data are presented as the mean ± S.D. and *p* values were calculated by two-sided Student's *t*-test.

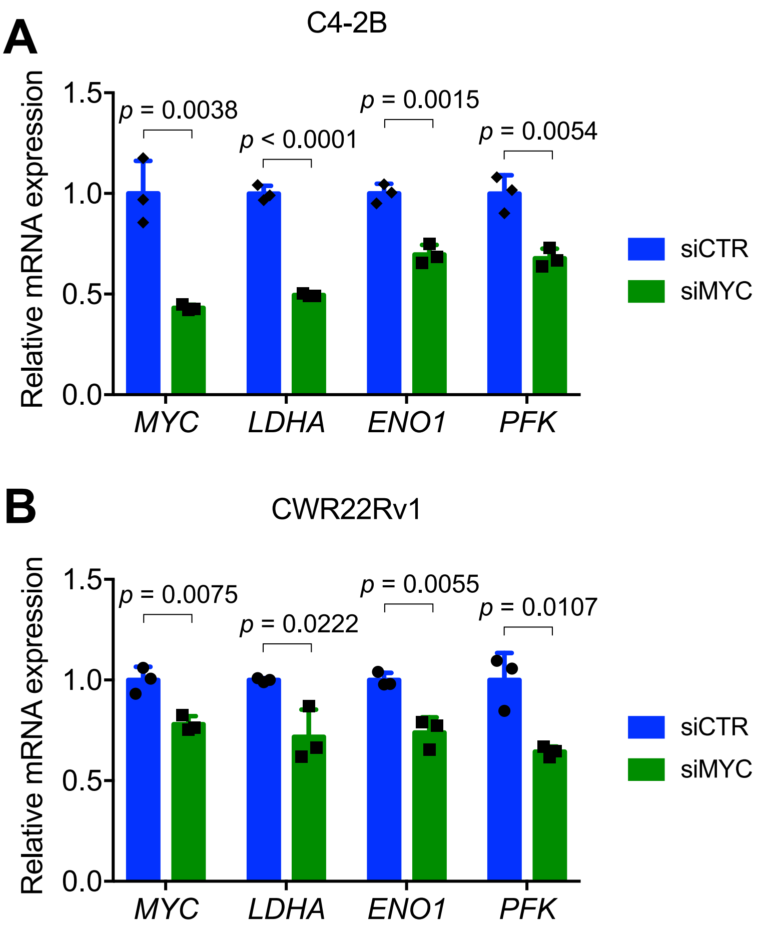

Figure S8. RNAi silencing of c-Myc reduces the expression of *LDHA, ENO1*, and *PFK*. (A–B) C4-2B **(A)** and CWR22Rv1 **(B)** cells were transfected with scramble siRNA (siCTR) or c-Myc siRNA (siMYC) and cultured for 24 h, followed by qRT-PCR analysis for *MYC*, *LDHA, ENO1*, and *PFK*. All data are presented as the mean ± S.D. and *p* values were calculated by two-sided Student's *t*-test.

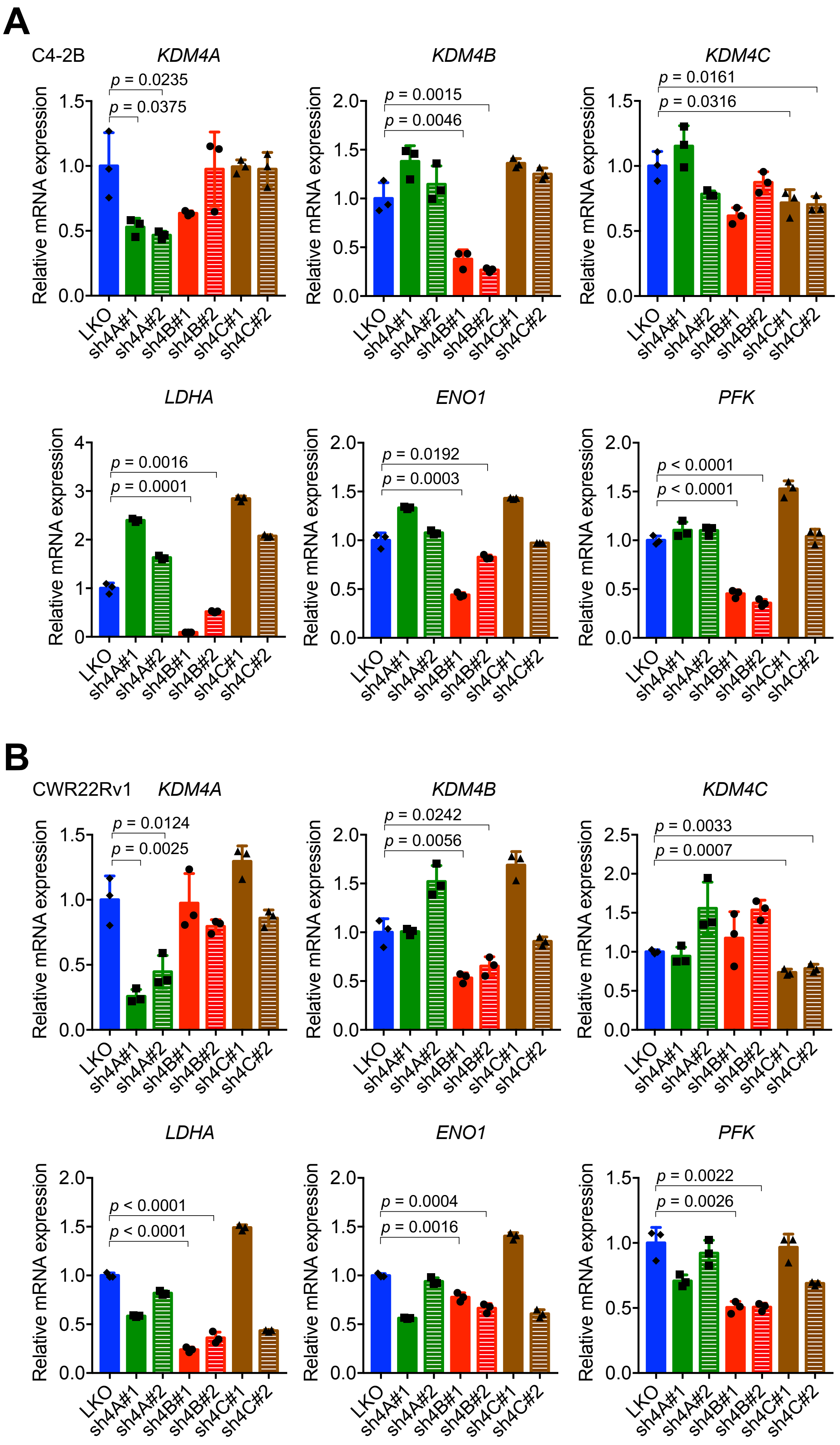
A
C4-2B
KDM4A
p = 0.0235
p = 0.0375
KDM4B
p = 0.0015
p = 0.0046
KDM4C
p = 0.0161
p = 0.0316
LDHA
p = 0.0016
p = 0.0001
ENO1
p = 0.0192
p = 0.0003
PFK
p < 0.0001
p < 0.0001
B
CWR22Rv1
KDM4A
p = 0.0124
p = 0.0025
KDM4B
p = 0.0242
p = 0.0056
KDM4C
p = 0.0033
p = 0.0007
LDHA
p < 0.0001
p < 0.0001
ENO1
p = 0.0004
p = 0.0016
PFK
p = 0.0022
p = 0.0026
Relative mRNA expression
LKO
sh4A#1
sh4A#2
sh4B#1
sh4B#2
sh4C#1
sh4C#2

Figure S9. Depletion of KDM4B, but not KDM4A and KDM4C, reduces the expression of *LDHA, ENO1*, and *PFK*. (A–B) The relative mRNA levels of KDM4-knockdown C4-2B **(A)** and CWR22Rv1 **(B)** cells. Cells were infected with lentivirus carrying control pLKO.1 (LKO), shKDM4A (sh4A#1 or sh4A#2), shKDM4B (sh4B#1 or sh4B#2), or shKDM4C (sh4C#1 or sh4C#2). Total RNA were prepared and subjected to qRT-PCR for *KDM4A*, *KDM4B*, *KDM4C*, *LDHA, ENO1*, and *PFK* expression. All data are presented as the mean ± S.D. and *p* values were calculated by two-sided Student's *t*-test.

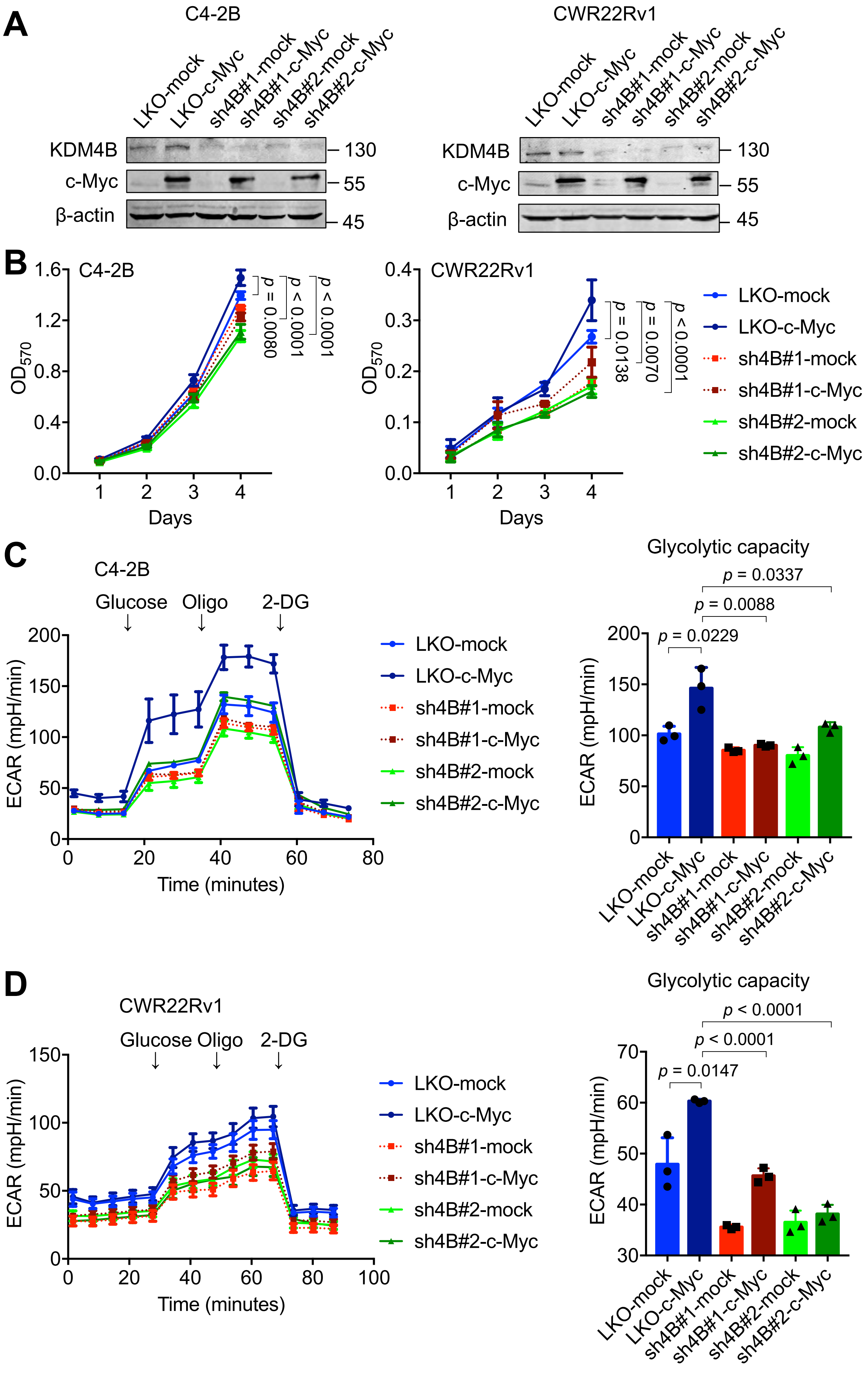
A
C4-2B
CWR22Rv1
LKO-mock
LKO-c-Myc
sh4B#1-mock
sh4B#1-c-Myc
sh4B#2-mock
sh4B#2-c-Myc
KDM4B
c-Myc
β-actin
130
55
45
B
OD570
Days
p = 0.0080
p < 0.0001
p < 0.0001
p = 0.0138
p = 0.0070
p < 0.0001
C
Glucose
Oligo
2-DG
ECAR (mpH/min)
Time (minutes)
Glycolytic capacity
p = 0.0337
p = 0.0088
p = 0.0229
D
p < 0.0001
p < 0.0001
p = 0.0147

Figure S10. KDM4B mediates the c-Myc-regulated EACR in CRPC cells. (**A**) Overexpression of c-Myc in KDM4B knockdown CRPC cells. C4-2B or CWR22Rv1 cells (LKO, sh4B#1, or sh4B#2) were transfected with a control vector (mock), or a c-Myc expressing vector (c-Myc) as indicated, followed by immunoblotting analysis. β-Actin was the internal control. **(B)** MTT cell proliferation assay of LKO, sh4B#1, and sh4B#2 C4-2B (left panel) and CWR22Rv1 (right panel) that introduced with either control (mock) or c-Myc expressed (c-Myc) vectors at indicated time points. (**C–D**) The ECARs of C4-2B (**C**) and CWR22Rv1 (**D**) cells were measured by the Seahorse analyzer. Cells (LKO, sh4B#1, and sh4B#2) were transfected with empty vector (mock), or c-Myc expressing vector (c-Myc). Adherent cells were incubated at glucose-free and $NaHCO_3$-free medium with subsequent injection of 10 mM glucose, 1 μM oligomycin (Oligo), and 50 mM 2-DG. The glycolytic capacity shown (right panel) was plotted from (**C** and **D**). All data are presented as the mean ± S.D. and *p* values were calculated by two-sided Student's *t*-test.

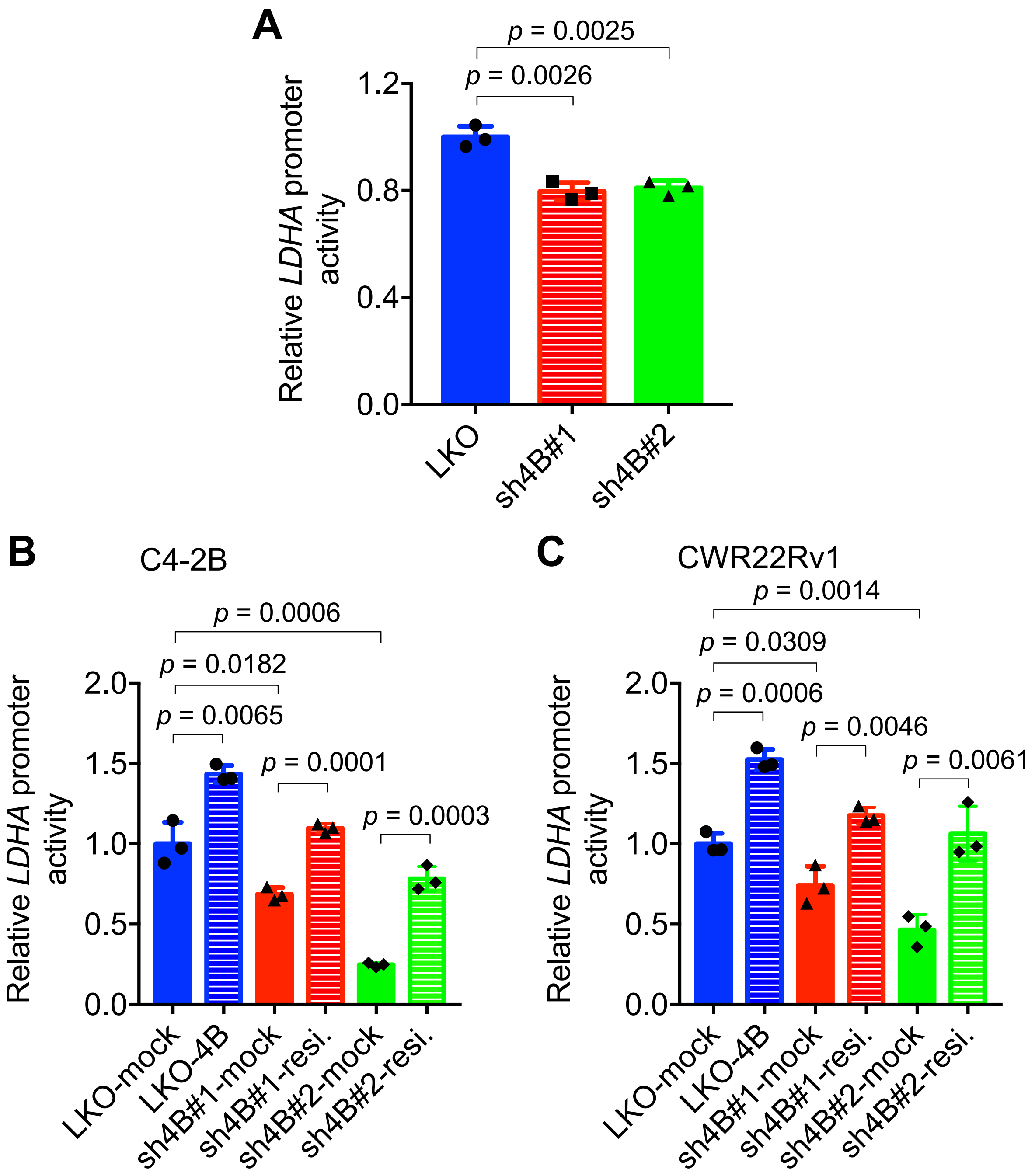

Figure S11. KDM4B promotes *LDHA* transactivation activity in CRPC cells. (A) CWR22Rv1 cells (LKO, sh4B#1, or sh4B#2) were co-transfected with a *LDHA* reporter construct and a β-galactosidase construct as an internal control, the activity was detected using Beta-Glo/One-Glo Assay System. **(B–C)** The *LDHA* promoter activity in KDM4B restored C4-2B **(B)** and CWR22Rv1 **(C)** cells. Cells (LKO, sh4B#1, and sh4B#2) were co-transfected with a *LDHA* reporter construct, a β-galactosidase construct as an internal control, and plus one of the empty vector (mock), KDM4B expression vector (4B), or specific shRNA-resistant KDM4B expression vector (resi.). The activity was detected using Beta-Glo/One-Glo Assay System. All data are presented as the mean ± S.D. and *p* values were calculated by two-sided Student's *t*-test.

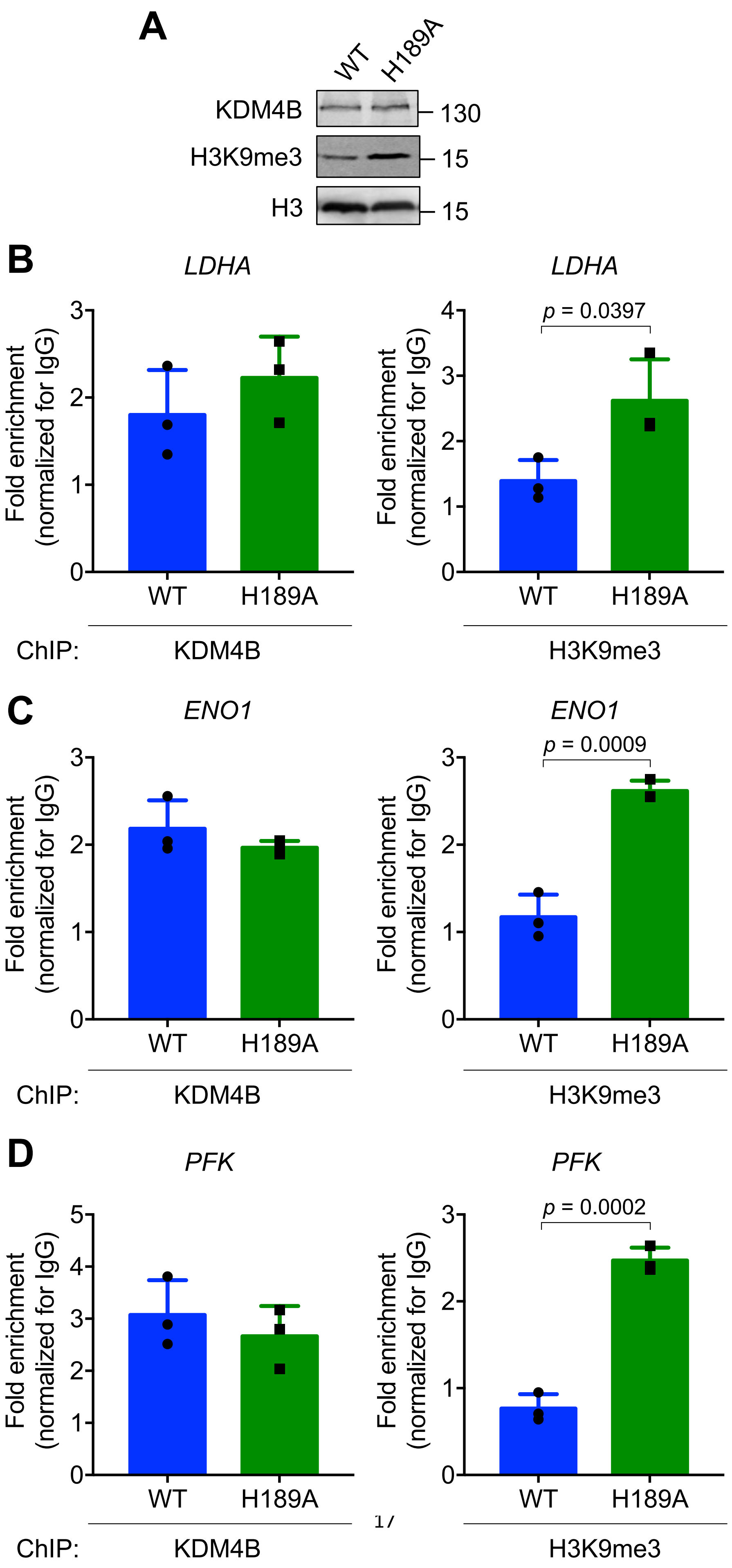

A
WT
H189A
KDM4B
H3K9me3
H3
130
15
15
B
LDHA
Fold enrichment (normalized for IgG)
p = 0.0397
WT
H189A
ChIP:
KDM4B
H3K9me3
C
ENO1
p = 0.0009
D
PFK
p = 0.0002

Figure S12. KDM4B-H189A binds to the loci of *LDHA, ENO1*, and *PFK* and fails to demethylate H3K9me3. (A) Overexpression of an inactive KDM4B mutant increases the H3K9me3 signal in C4-2B. C4-2B cells were transfected with a wild-type (WT) or an inactive mutant H189A of KDM4B for 24 h, followed by immunoblotting using an antibody as indicated. β-Actin was the internal control. **(B–D)** Chromatin immunoprecipitation (ChIP) analysis of KDM4B and H3K9me3 occupancy on the *LDHA* **(B),** *ENO1* **(C)**, and *PFK* **(D)** promoter loci. ChIP was performed with specific antibodies against KDM4B and H3K9me3 in WT or H189A. ChIP DNAs were analyzed by qPCR with primers that amplify specific genomic regions. Data were presented as the average of fold enrichment normalized to IgG ± SD from three independent experiments. Statistical analysis was assessed by two-sided Student's *t*-test.

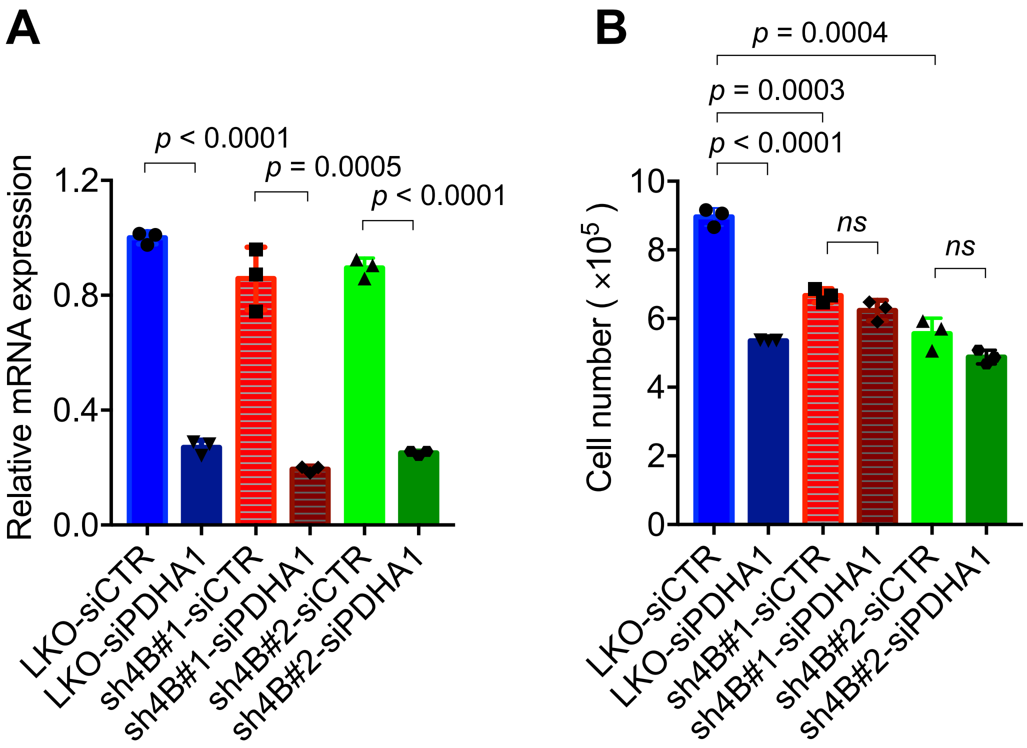

Figure S13. The depletion of PDHA1 does not restore the proliferation suppressed by KDM4B knockdown in CWR22Rv1. (A) PDHA1 depletion in CWR22Rv1 is validated by qRT-PCR analysis. Control (LKO) and KDM4B-knockdown (sh4B#1 and sh4B#2) CWR22Rv1 cells transfected with scramble siRNA (siCTR) or PDHA1 siRNA (siPDHA1) were subjected to qRT-PCR analysis. Data are normalized to LKO-siCTR. **(B)** Cell proliferation of PDHA1-depleted CWR22Rv1. CWR22Rv1 (LKO, sh4B#1, or sh4B#2) was treated with scramble siRNA (siCTR) or PDHA1 siRNA (siPDHA1) and cultured for 72h, followed by cell-number quantification. All data are presented as the mean ± S.D. p values were calculated by two-sided Student's *t*-test.

Supplementary table

Table S1. Primer sequences used in this study		
Gene	Forward (5' to 3')	Reverse (5' to 3')
qRT-PCR		
KDM4A	GCCGCTAGAAGTTTCAGTGAG	GCGTCCCTTGGACTTCTTATT
KDM4B	CTTCACGCAGTACAATATCC	CGTCGTCATCATACAAAGAG
KDM4C	ACGCGAGTATCTTTCCCCTC	CGCGCTGTGGTTAACTTAGG
MYC	AATGAAAAGGCCCCCAAGGTAGTTATCC	GTCGTTTCCGCAACAAGTCCTCTTC
GLUT1	GATTGGCTCCTTCTCTGTGG	TCAAAGGACTTGCCCAGTTT
HK2	GATTGTCCGTAACATTCTCATCGA	TGTCTTGAGCCGCTCTGAGAT
PFK	AGATGCGCACCAGCATCAACG	GAACCCGGCACATTGTTGGA
ALDOA	ATGCCCTACCAATATCCAGCA	GCTCCCAGTGGACTCATCTG
GAPDH	CATCATCCCTGCCTCTACT	TGCTTCACCACCTTCTTG
PGM1	CCTTCGCGCTCGCTCTTCTG	TAACCAAGCGACCGATCTTTCCTG
ENO1	GTGTGGCTCTAACCCTCTGG	TCTGTGACGTTCAGTTTCTTGC
PKM2	GAACTTCTCTCATGGAACTCAT	GATCTCAGGTCCTTTAGTGTCTA
LDHA	AGCTGTTCCACTTAAGGCCC	TGGAACCAAAAGGAATCGGGA
PDK1	GAGAGCCACTATGGAACACCA	GGAGGTCTCAACACGAGGT
PDK2	TCGTATAGGCTTGCACCCTG	CCTCCCTTAACGGGTCTTGG
PDK3	GTGAACCCAGGGATGCTTCA	GGACCACAGCATTCAGTCCT
IDH2	GAAGGTGTGCGTGGAGAC	CCGTGGTGTTCAGGAAGT
KGDH	TTGGCTGGAAAACCCCAAAAG	TGTGCTTCTACCAGGGACTGT
SUCLG1	GAGCAACGGCTTCTGTCATTT	TGCTTGACTCGTACCATGTCC
SDHA	TGGGAACAAGAGGGCATCTG	CCACCACTGCATCAAATTCATG
MDH1	TTTGGATCACAACCGAGCTAAAG	ACATCTGGATACTGAGTCGAGG
GLS1	GACATGGAACAGCGGGACTAT	TGTCCTTGGGGAAAGGGTTT
PCK1	GATGCAGGGGTCCGGTTTATT	GAAGCCGTAGGTGTTGGAGA
PCK2	GGCATTCGAGATTTTGTAGAG	CAGCAGTTATTGTACTTGGG
PDHA1	GGGACGTCTGTTGAGAGAGC	TGTGTCCATGGTAGCGGTAA
18S	AAACGGCTACCACATCCAAG	CCTCCAATGGATCCTCGTTA
ChIP		
LHDA	AGCTGTTCCACTTAAGGCCC	CTACCGTACCGGGAATGCAC
ENO1	GGAGATCTCGCCGGCTTTAC	AGCCCTTCCCCAATCATTACC
PFK	GGGGATGCTCAAGGTGCG	CTCTTCTCCCCGGTTCTCC

Table S1. Primer sequences used in this study (continued)		
Gene	Forward (5' to 3')	Reverse (5' to 3')
c-Myc expression vector construction		
c-Myc-ΔN	CACAAGGATCCATGCCCCTCAACGTTAGCTTC	GTTGTCTCGAGTTACGAGGCGCAGAGCTTG
c-Myc-ΔC	CACCGAACGGATCCCAAGACTCCAGCGCCTTC	GTGGGCTCGAGTTACGCACAAGAGT
LDHA-Luc construction		
LDHA-Luc	AAAAAGGTACCGAATCAGCAAGAATACAGG	AAAAAGCTAGCCTTCTGGAAAGCGGCT
